# Hybrid Cubature Kalman filtering for identifying nonlinear models from sampled recording: Estimation of neuronal dynamics

**DOI:** 10.1371/journal.pone.0181513

**Published:** 2017-07-20

**Authors:** Mahmoud K. Madi, Fadi N. Karameh

**Affiliations:** Department of Electrical and Computer Engineering, American University of Beirut, Beirut, Lebanon; Brown University, UNITED STATES

## Abstract

Kalman filtering methods have long been regarded as efficient adaptive Bayesian techniques for estimating hidden states in models of linear dynamical systems under Gaussian uncertainty. Recent advents of the Cubature Kalman filter (CKF) have extended this efficient estimation property to nonlinear systems, and also to hybrid nonlinear problems where by the processes are continuous and the observations are discrete (continuous-discrete CD-CKF). Employing CKF techniques, therefore, carries high promise for modeling many biological phenomena where the underlying processes exhibit inherently nonlinear, continuous, and noisy dynamics and the associated measurements are uncertain and time-sampled. This paper investigates the performance of cubature filtering (CKF and CD-CKF) in two flagship problems arising in the field of neuroscience upon relating brain functionality to aggregate neurophysiological recordings: (i) estimation of the firing dynamics and the neural circuit model parameters from electric potentials (EP) observations, and (ii) estimation of the hemodynamic model parameters and the underlying neural drive from BOLD (fMRI) signals. First, in simulated neural circuit models, estimation accuracy was investigated under varying levels of observation noise (SNR), process noise structures, and observation sampling intervals (*dt*). When compared to the CKF, the CD-CKF consistently exhibited better accuracy for a given SNR, sharp accuracy increase with higher SNR, and persistent error reduction with smaller *dt*. Remarkably, CD-CKF accuracy shows only a mild deterioration for non-Gaussian process noise, specifically with Poisson noise, a commonly assumed form of background fluctuations in neuronal systems. Second, in simulated hemodynamic models, parametric estimates were consistently improved under CD-CKF. Critically, time-localization of the underlying neural drive, a determinant factor in fMRI-based functional connectivity studies, was significantly more accurate under CD-CKF. In conclusion, and with the CKF recently benchmarked against other advanced Bayesian techniques, the CD-CKF framework could provide significant gains in robustness and accuracy when estimating a variety of biological phenomena models where the underlying process dynamics unfold at time scales faster than those seen in collected measurements.

## Introduction

Physiological signal recordings have long played a central role in probing and deciphering the functional state of the underlying biological process. Towards this goal, dynamical system modeling aims principally to develop a causal link between the observed signals and the predicted process outputs. In brain sciences, modeling is generally intended to provide a link between the ongoing activity of a neuronal system and a host of associated aggregate recordings including directly related electrical measurements (such as the electroencephalogram EEG, electrical corticogram ECoG, Local Field Potentials LFP) and indirectly related metabolic measurements (such as fMRI and SPECT). For a vast majority of these models, the computational and identification complexity of these models quickly increases with the inclusion of realistic assumptions on both the process and its measurement conditions.

First, at the process level, realistic descriptions often result in continuous-time, nonlinear, stochastic and possibly time-varying dynamics. Starting with a set of ordinary differential equations, models commonly include (i) nonlinear relationships among several variables (e.g. voltage-dependent ionic conductances), (ii) uncertainty or randomness in describing the process response to its environment (e.g. in vivo synaptic noise), and (iii) modulation of the process itself by external inputs or factors (e.g. effect of neuromodulators ACh). Second, at the measurement level, observation of the process is attained indirectly through one or more continuous-time variables that relate to the neuronal activity and are limited by spatial smearing (e.g. extracellular currents) or temporal filtering (e.g. blood oxygenation levels). Although both of the underlying processes are continuous, the temporal bandwidth of the hemodynamic process is considerably smaller than that of the neuronal dynamics (or its output contains much lower frequencies) and hence can be recorded at much larger time intervals (Nyquist rate). Accordingly, the relative time scale of the recording devices for those activities make the neuronal activity well described by a continuous process and hemodynamic activity by a time-sampled continuous process, or a discrete process. In other words, and by taking “snapshots” or images of the hemodynamic process at regular intervals in time, the observations constitute a sequence of noisy discrete-time physiological recordings.

Along with the increase in model complexity, the correct identification of the model parameters and the accurate estimation of its hidden internal states become key challenges. This is particularly true since the efficiency and performance of available estimation tools depend on a set of assumptions on the process dynamics (linearity, time-invariance) and its operating conditions (process and measurement noise structure) that become clearly violated in these models.

From a system theoretic viewpoint, state space formulations constitute a flexible framework whereby both the modeling and estimation problems can be combined for a wide range of realistic physiological modeling assumptions. In the context of modeling, state space allows for separate descriptions of the dynamic processes and their uncertainties (continuous-time dynamics and noise impact in both the hidden states and observation variables) from the method of observation and its imperfections (discrete time noisy multiple channel recordings) [1]. In the context of estimation, state space summarizes the system history in a set of first order dynamics memory elements (or states) whereby knowledge of their current value and future inputs completely characterizes the system evolution into the future (first order Markov), thereby allowing for efficient time-recursive estimation.

This type of state estimation problems is usually solved with Bayesian filters whereby the posterior probability density function (pdf) of the states is constructed, based on all available information up to current time, to provide a complete statistical description of the states at current time [2]. Subsequently, new information that becomes available from new measurements is combined with the old information to modify the posterior pdf using Bayes theorem.

Arguably, the Kalman filter is the most widely used type of Bayesian filters available to solve the state estimation problem. In the Kalman setup, it is assumed that both the state noise and the measurement disturbance are samples of an additive, zero mean random processes that admit Gaussian probability distributions [3].

The Kalman filter is a recursive estimation filter since the posterior densities of the states are updated with new measurement without the need to reprocess all previous measurement data. The filter consists mainly of two steps, the prediction step and the update step, that are also commonly referred to as the time update and the measurement update, respectively. The prediction step involves the computation of the mean and covariance matrix of the predictive state pdf using the process model and the estimated state pdf from the previous time step. Then, the update step employs Bayes theorem to modify the predictive pdf using information available from the new measurement. As an adaptive technique, therefore, a Kalman filter allows for predicting time variations in the estimated densities.

Strictly speaking, the Kalman filter is an optimal Bayesian estimator only for linear systems because it updates the first and second order moments of linear combinations of Gaussian distributed random processes, which are also Gaussian. Extensions of the Kalman filter were introduced in the past in order to deal with practical systems that involve nonlinearities. Such filters are the Extended Kalman filter (EKF) [4], the Unscented Kalman filter (UKF) [5–7] and the most recently introduced filter the Cubature Kalman filter (CKF) [2, 8].

Kalman filter extensions (EKF, UKF, and CKF) that deal with nonlinear systems have also found their way to numerous applications in a wide variety of areas in Biology [9–15]. In the neural sciences, KF applications include neural activity modeling and estimation [10, 16–18] motor activity decoding for neural prosthesis [19, 20], intracranial pressure estimation [21], Seizure prediction and control [22–26], Sleep and EEG modeling [27–30], and particularly brain connectivity estimation in psychology and cognition [31–35]. The time-varying adaptive nature of Kalman filtering continues to place it among other popular estimation techniques, particularly those based on Dynamic Causal Modelling (DCM) [36]. DCM is based on generative models that are compared within a Bayesian framework in order to infer the functional connectivity between neuronal populations or brain regions from observed data (EEG, MEG, or fMRI) [37–40].

The Cubature Kalman filter is designed for the estimation of hidden or unobserved states in a nonlinear dynamical system that is subjected to additive Gaussian noise. The CKF was originally introduced as an approximate Bayesian filter for discrete-time nonlinear filtering problems whereby the predictive density of the joint state-measurement random variable is assumed to be Gaussian. In this way, the optimal Bayesian filter reduces to the problem of computing various multi-dimensional Gaussian-weighted moment integrals, with numerical approximations obtained by invoking a third-degree spherical-radial cubature rule.

As presented above, representations of physiological processes, such as neural systems, often admit state-space models that are of the continuous-discrete hybrid types. For estimation of these systems, therefore, the hybrid Continuous-Discrete Cubature Kalman Filter (CD-CKF), introduced as an extension of the CKF for mixed systems, seems to be more natural. The CD-CKF discretizes the continuous process equation in SDE form using the Itô-Taylor expansion of order 1.5 and transforms it to stochastic difference equation in discrete time. This transformation will result in a state-space model with both process and measurement equations expressed in stochastic difference equations in discrete time.

In the paper, we address the accuracy of state and parameter estimation using CKF and CD-CKF techniques in the context of neural state estimation from EEG and fMRI recordings as specific examples of physiological dynamical system modeling. Starting with nonlinear state-space simulation models, we elaborate estimation performance while varying conditions related to (i) the observation sampling frequency, (ii) the observation signal-to-noise ratio and (iii) the structure of the additive noise process underlying the state dynamics. In particular, we aim to highlight those situations where an added benefit can be obtained by explicitly employing a hybrid filtering. We pay specific attention to the effect of the sampling interval of the observations principally because it relates to the inherent time constants (speed of dynamics) of the underlying continuous processes and hence constrains the modeler’s ability to recover detailed dynamics from observations obtained using a given recording modality. In the case of estimating neural activity, electrical potential recordings (EEG, MEG) theoretically have a real-time accuracy since these are manifestations of the underlying electrical neural dynamical activity. Imaging modalities (fMRI, SPECT), on the other hand, have a much lower time resolution because these reflect observations of slow continuous-time metabolic processes that are indirectly related to the fast underlying neural activity. Specifically for fMRI, the time sampling interval of the blood oxygenation levels is on the order of one second [41], which is well beyond the milliseconds details of the dynamics of synaptic activity. Thus, with realistic recording conditions, it is suspected that the CD-CKF might be superior in cases where one aims to recover hidden process dynamics from low-pass filtered indirect observations, such as when inferring neural activity drive from fMRI data as a determinant for estimating functional connectivity in brain networks.

We also compare the accuracy of the CD-CKF and CKF techniques in estimating the neural activity and parameters for simulated neural models in cases where the observation signal-to-noise ratio are decreased, and/or the Gaussian process noise assumptions are violated.

Low signal to noise ratios are common for modalities that record electrical potentials at a location distant from the source (depth electrodes, scalp EEG) due to spatial filtering (smearing (~0.5 cm^2^) and activity aggregation across numerous neural subtypes (leading to unmodeled signal components).

Finally, and since Kalman-based techniques invariably assume that process noise is of Gaussian nature (for a continuous time, the derivative of the state is driven by a Wiener process), we aim to assess, using Monte-Carlo simulations, the performance of both CKF and CD-CKF when noise structures violate Gaussianity. In neural estimation, non-Gaussian noise models from are common. Examples include the additive noise in synaptic dynamics (approximating in vivo conductance fluctuations) that has specific structures (Ornstein–Uhlenbeck process [42], and the afferent neural activity impinging onto a given population, that has been reported to possess an un-symmetric tailed distribution [43–48].

We note here that performance assessment is limited here to improvements of hybrid CD-CKF over the CKF estimator—with the understanding that the utility of the CKF in solving these problems was earlier benchmarked against many other state-of-the-art estimation techniques. In the area of neural modeling, Havlicek et al. (2011) [32] validated the CKF algorithm against a main contender, namely the dynamic expectation maximization that used variational Bayesian techniques [49]. The authors demonstrated that, for hemodynamic models of evoked brain responses in fMRI, marked improvements can be obtained using CKF estimation when compared to the dynamic expectation maximization method (DEM) in terms of inversion of nonlinear dynamic models, including estimation of the states, input and parameters. In addition, and because DEM requires embedding of the states while a CKF does not, the CKF was computationally more efficient in preforming model inversion (see [32] for more details).

The paper is organized as follows. In the next section, we present the Kalman filtering formulations for the CKF and CD-CKF. We then introduce a simplified model of continuous neural activity (NA) dynamics and associated electric potentials (EP) recordings. A state-space formulation of this model under different additive noise structures is subsequently introduced as part of the Kalman filtering setup. We similarly introduce the hemodynamic model linking neural drive (output) to recorded BOLD signals under fMRI as well as a joint neuronal-hemodynamic model. Next, we compare the performance of CKF and CD-CKF in state estimation accuracy from simulated EP observations under different assumptions on (a) observation data sampling rate, (b) observation signal-to-noise ratio and (c) process noise structures. We then contrast performance of the two filters in estimating neural drive and parameters in the hemodynamic model and show some preliminary results on the joint neuronal-hemodynamic model. Finally, we summarize the findings and discuss their significance and implications in physiological process estimation.

## Methods

### Neuronal model description

In this section, we will introduce the mathematical description of the simulation models for neural activity (NA) dynamics. We will adopt a simplified neuronal model to describe the neuronal dynamics of different populations in a cortical column. The model incorporates active neurotransmitter-gated synaptic processes [50]. The dynamics of the membrane potential are formulated as a parallel RC circuit where capacitive synaptic current flow balances the sum of all currents across the membrane [50]. The dynamics of the membrane potential are given by the following stochastic differential equations:

$$C\dot{V}=g_{L}\left(V_{L}-V\right)+g_{E}\left(V_{E}-V\right)+g_{I}\left(V_{I}-V\right)+I+\Gamma_{V}$$

Where C is the membrane capacitance, V is the membrane potential, I is the input current, *Γ*_*V*_ is a Gaussian noise, and the currents across the membrane are as follows (see Table 1):

- *g*_*E*_(*V*_*E*_ − *V*): is the excitatory sodium (Na^+^) current with conductance *g*_*E*_ and reversal potential *V*_*E*_.
- *g*_*I*_(*V*_*I*_ − *V*): is the inhibitory chloride (Cl^−^) current with conductance *g*_*I*_ and reversal potential *V*_*I*_.
- *g*_*L*_(*V*_*L*_ − *V*): is the potassium (K^+^) leak current with conductance *g*_*L*_ and reversal potential *V*_*L*_.

**Table 1:** Model parameters.

Parameter	Physiologic interpretation	Value	Unit
***V***^***i***^	Membrane potential, *i* = 1, 2, 3 for granular, supra-granular, and infra-granular respectively.	---	mV
***V***_***L***_	potassium (K^+^) reversal potential	-70	mV
***V***_***E***_	sodium (Na^+^) reversal potential	60	mV
***V***_***I***_	chloride (Cl^−^) reversal potential	-90	mV
***V***_***R***_	Threshold potential	-40	mV
***C***	Membrane capacitance	10	μF
***I***	Input current at the granular layer	---	μA
***g***_***L***_	Conductance of potassium leak current	1	mS
$g_{E}^{i}$	Excitatory sodium current conductance, *i* = 1, 2, 3 for granular, supra-granular, and infra-granular respectively.	---	mS
$g_{I}^{i}$	Inhibitory chloride current conductance, *i* = 1, 2, 3 for granular, supra-granular, and infra-granular respectively.	---	mS
***κ***_***E***_	Sodium diffusion rate constant (opening of sodium channels)	0.25	ms^-1^
***κ***_***I***_	Chloride diffusion rate constant (opening of chloride channels)	0.0625	ms^-1^
***α***	Constant that controls the voltage sensitivity of the activation function	0.56	---
$\gamma_{2,1}^{I}$	Inhibitory connection strength from supra-granular to granular layers	0.7	---
$\gamma_{2,3}^{I}$	Inhibitory connection strength from supra-granular to infra-granular layers	2	---
$\gamma_{2,2}^{I}$	Inhibitory connection strength within the supra-granular layer	0.25	---
$\gamma_{3,1}^{E}$	Excitatory connection strength from infra-granular to granular layers	0.5	---
$\gamma_{3,2}^{E}$	Excitatory connection strength from infra-granular to supra-granular layers	1	---
$\gamma_{1,3}^{E}$	Excitatory connection strength from granular to infra-granular layers	1	---

The conductances can also be described by stochastic differential equations whose dynamics depend on the pre-synaptic input (*ς*) and a characteristic rate constant (*κ*).

$${\dot{g}}_{i}=\kappa_{i}\left(\varsigma_{i}-g_{i}\right)+\Gamma_{i}$$

Where *g*_*i*_(i = E, I) represent the excitatory and inhibitory conductance, and *Γ*_*i*_ is Gaussian noise.

The pre-synaptic input to a given neuron, denoted by $\varsigma_{\text{l}}^{\left(\text{i}\right)}$, is the firing rate in another neuron j times a coupling parameter *γ*^(*j*,*i*)^ [51]:

$$\varsigma_{l}^{\left(i\right)}=\gamma_{l}^{\left(j,i\right)}\sigma \left(V^{\left(j\right)}-V_{R}\right)$$

Where *σ*(.) is a sigmoid activation function that transforms the postsynaptic potential of neuron j to firing rate, and is given by [52]:

$$\sigma \left(V^{\left(j\right)}-V_{R}\right)=\frac{1}{1+e^{-\alpha \left(V^{\left(j\right)}-V_{R}\right)}}$$

Where *V*_*R*_ is a threshold potential, and *α* is a constant that determines the slope (voltage sensitivity) of the activation function.

**Fig 1:**
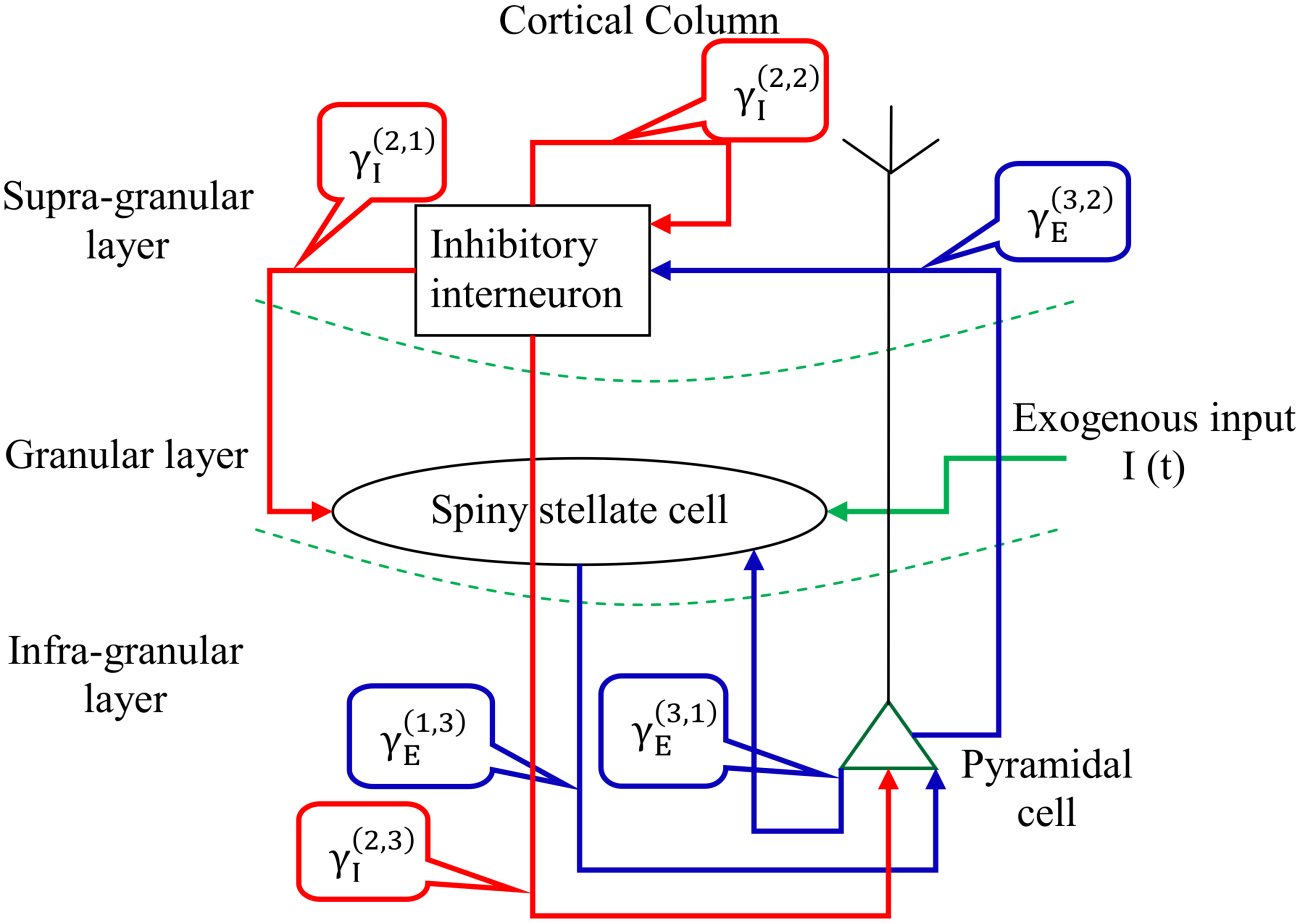
Cortical column architecture. A cortical column is segregated into three layers where the input granular layer is composed of spiny stellate cells, the supra-granular layer is composed of inhibitory interneurons, and the output infra-granular layer is composed of pyramidal cells. Intrinsic connections between layers are illustrated with arrows: red arrows are inhibitory, and blue arrows are excitatory.

For simplicity, we will consider a cortical column that is composed of three layers (Fig 1):

1. The granular layer: consists of excitatory spiny stellate cells.
2. The supra-granular layer: consists of inhibitory interneurons.
3. The infra-granular layer: consists of excitatory pyramidal cells.

The model described above will be adopted to describe the stochastic dynamics of interacting populations in a cortical column. Thus, for each population *i* = 1, 2, 3:

$$C{\dot{V}}^{\left(i\right)}=g_{L}\left(V_{L}-V^{\left(i\right)}\right)+g_{E}^{\left(i\right)}\left(V_{E}-V^{\left(i\right)}\right)+g_{I}^{\left(i\right)}\left(V_{I}-V^{\left(i\right)}\right)+I+\Gamma_{V}$$

$${\dot{g}}_{E}^{\left(i\right)}=\kappa_{E}\left(\varsigma_{E}^{\left(i\right)}-g_{E}^{\left(i\right)}\right)+\Gamma_{E}$$

$${\dot{g}}_{I}^{\left(i\right)}=\kappa_{I}\left(\varsigma_{I}^{\left(i\right)}-g_{I}^{\left(i\right)}\right)+\Gamma_{I}$$

$$\varsigma_{l}^{\left(i\right)}=\gamma_{l}^{\left(j,i\right)}\sigma \left(V^{\left(j\right)}-V_{R}\right),\;l=E,I$$

Where the input *I* is at the granular layer (population 1).

These stochastic differential equations can be formulated in state-space model of the form:

$$\dot{x}=f\left(x,I\right)+\Gamma$$

Where the state vector *x* comprises the membrane potentials, the excitatory and inhibitory conductance, and *f*(.) is a vector that comprises the equations of motion of each state:

$$x={\left[\begin{array}{ccccc} V^{\left(1\right)} & g_{I}^{\left(1\right)} & g_{E}^{\left(1\right)} & \begin{array}{ccc} V^{\left(2\right)} & g_{I}^{\left(2\right)} & g_{E}^{\left(2\right)} \end{array} & \begin{array}{ccc} V^{\left(3\right)} & g_{I}^{\left(3\right)} & g_{E}^{\left(3\right)} \end{array} \end{array}\right]}^{T}$$

$$f\left(x,I\right)=\left[\begin{array}{c} \frac{1}{C}\left(g_{L}\left(V_{L}-V^{\left(1\right)}\right)+g_{E}^{\left(1\right)}\left(V_{E}-V^{\left(1\right)}\right)+g_{I}^{\left(1\right)}\left(V_{I}-V^{\left(1\right)}\right)+I\right)\\ \kappa_{I}\left(\gamma_{I}^{\left(2,1\right)}\sigma \left(V^{\left(2\right)}-V_{R}\right)-g_{I}^{\left(1\right)}\right)\\ \kappa_{E}\left(\gamma_{E}^{\left(3,1\right)}\sigma \left(V^{\left(3\right)}-V_{R}\right)-g_{E}^{\left(1\right)}\right)\\ \frac{1}{C}\left(g_{L}\left(V_{L}-V^{\left(2\right)}\right)+g_{E}^{\left(2\right)}\left(V_{E}-V^{\left(2\right)}\right)+g_{I}^{\left(2\right)}\left(V_{I}-V^{\left(2\right)}\right)\right)\\ \kappa_{I}\left(\gamma_{I}^{\left(2,2\right)}\sigma \left(V^{\left(2\right)}-V_{R}\right)-g_{I}^{\left(2\right)}\right)\\ \kappa_{E}\left(\gamma_{E}^{\left(3,2\right)}\sigma \left(V^{\left(3\right)}-V_{R}\right)-g_{E}^{\left(2\right)}\right)\\ \frac{1}{C}\left(g_{L}\left(V_{L}-V^{\left(3\right)}\right)+g_{E}^{\left(3\right)}\left(V_{E}-V^{\left(3\right)}\right)+g_{I}^{\left(3\right)}\left(V_{I}-V^{\left(3\right)}\right)\right)\\ \kappa_{I}\left(\gamma_{I}^{\left(2,3\right)}\sigma \left(V^{\left(2\right)}-V_{R}\right)-g_{I}^{\left(3\right)}\right)\\ \kappa_{E}\left(\gamma_{E}^{\left(1,3\right)}\sigma \left(V^{\left(1\right)}-V_{R}\right)-g_{E}^{\left(3\right)}\right) \end{array}\right]$$

### State-Space model

The model described above in stochastic differential equations form can be formulated in state-space model of the form:

$$\text{Process Equation}:\;\dot{x}\left(t\right)=f\left(x,I\right)+\Gamma$$

$$\text{Measurement Equation}:\;z_{k}=h\left(x_{k},k\right)+w_{k}$$

Where $x\;\in \;{\mathbb{R}}^{n}$ is the state vector of the dynamic system at time t, *I* is the exogenous input, $z_{k}\;\in \;{\mathbb{R}}^{d}$ is the measurement at discrete time *t*_*k*_, $f:\;{\mathbb{R}}^{n}\times \mathbb{R}\to {\mathbb{R}}^{n}$ is the drift coefficient, $h:\;{\mathbb{R}}^{n}\times \mathbb{R}\to {\mathbb{R}}^{d}$ is the measurement function, $\Gamma \;\in \;{\mathbb{R}}^{n}$ and $w_{k}\;\in \;{\mathbb{R}}^{d}$ are vectors of zero mean random Gaussian noise.

The activity of infra-granular layer is considered as the output layer in which its activity is observed and serves as a measurement. Our EP recording is assumed to be a simple linearized filtering of the voltages of infra-granular layer. Thus, the measurement equation function *h*(*x*_*k*_, *k*) depends on the infra-granular layer membrane potentials (*V*^(3)^). We will consider as an output measure of this cortical column the value of infra-granular membrane potential at a given discrete time *k*.

$$h\left(x_{k},k\right)=V_{k}^{\left(3\right)}$$

Where $V_{k}^{\left(3\right)}$ represents infra-granular membrane potential at discrete time instant *k*.

### Model dynamics

An exogenous input arrives at the granular level and excites the spiny stellate cells, which in turns send postsynaptic excitation to pyramidal neurons located in the infra-granular layer.

The activated pyramidal cells send a feedback signal to both granular and supra-granular layers, where the inhibitory interneurons in supra-granular layer tend to inhibit both granular and infra-granular cells as well as the interneurons themselves.

The activity of infra-granular layer is considered as the main evoked output in a cortical column [53]. Thus, this layer will serve as an output layer in which its activity is observed and serve as measurement in the Kalman setup.

**Fig 2:**
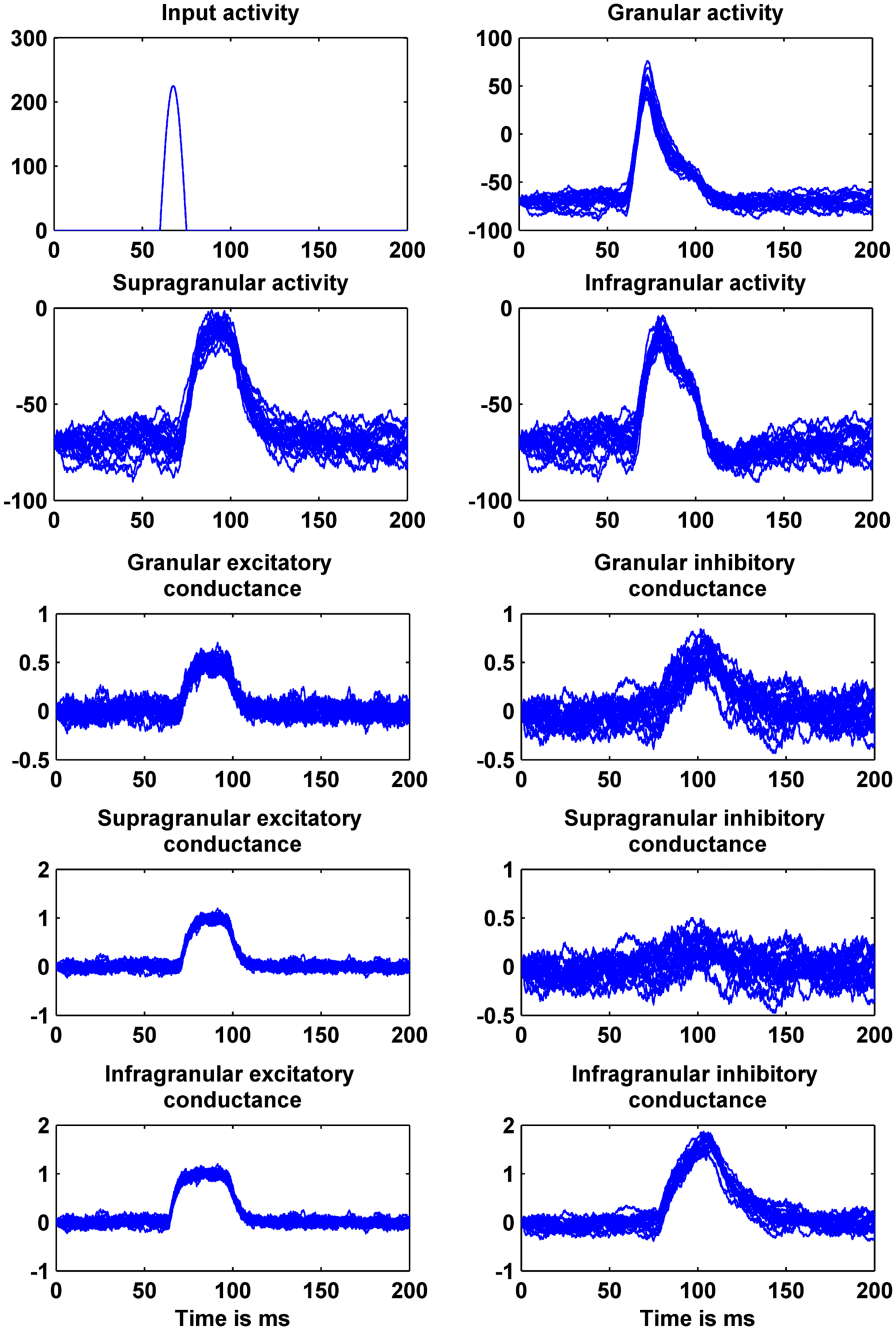
Cortical model activity for all states at different layers. The exogenous input is shown in the top left panel for the case where the additive noise is white.

To illustrate the basic network dynamics, we examined the neuronal responses of this network (shown in Fig 1) for a given afferent input by integrating the equations aforementioned using IT-1.5 discretization method. We considered 20 realizations to demonstrate the effect of stochastic fluctuations of the additive white noise on the behavior of this network (Fig 2).

### Colored noise

We will describe the model for the case where the additive noise is colored noise. The differential equations are formulated in state-space model of the form:

$$\dot{x}\left(t\right)=f\left(x,I\right)+\Psi$$

Where **Ψ** is filtered white noise.

In order to be able to simulate this model with colored noise using the IT-1.5 discretization method for SDE, we augmented the state vector to include the colored noise as state variables driven by white noise. The augmented state vector becomes:

$$x^{aug}={\left[\begin{array}{cc} x & \Psi \end{array}\right]}^{T}$$

$$x={\left[\begin{array}{ccccc} V^{\left(1\right)} & g_{I}^{\left(1\right)} & g_{E}^{\left(1\right)} & \begin{array}{ccc} V^{\left(2\right)} & g_{I}^{\left(2\right)} & g_{E}^{\left(2\right)} \end{array} & \begin{array}{ccc} V^{\left(3\right)} & g_{I}^{\left(3\right)} & g_{E}^{\left(3\right)} \end{array} \end{array}\right]}^{T}$$

$$\Psi ={\left[\begin{array}{ccccc} \begin{array}{c} \Psi_{V^{\left(1\right)}} \end{array} & \Psi_{g_{I}^{\left(1\right)}} & \Psi_{g_{E}^{\left(1\right)}} & \begin{array}{ccc} \Psi_{V^{\left(2\right)}} & \Psi_{g_{I}^{\left(2\right)}} & \Psi_{g_{E}^{\left(2\right)}} \end{array} & \begin{array}{ccc} \Psi_{V^{\left(3\right)}} & \Psi_{g_{I}^{\left(3\right)}} & \Psi_{g_{E}^{\left(3\right)}} \end{array} \end{array}\right]}^{T}$$

Using the augmented vector notation, the system now is a state-space model described as stochastic differential equations:

$${\dot{x}}^{aug}=f^{aug}\left(x^{aug},I\right)+\Theta \Gamma$$

$$\left[\begin{array}{c} \dot{x}\\ \dot{\Psi } \end{array}\right]=\left[\begin{array}{c} f\left(x,I,\Psi \right)\\ f\left(\Psi \right) \end{array}\right]+\Theta \Gamma$$

$$f\left(x,I,\Psi \right)=\left[\begin{array}{c} \frac{1}{C}\left(g_{L}\left(V_{L}-V^{\left(1\right)}\right)+g_{E}^{\left(1\right)}\left(V_{E}-V^{\left(1\right)}\right)+g_{I}^{\left(1\right)}\left(V_{I}-V^{\left(1\right)}\right)+I\right)+\Psi_{V^{\left(1\right)}}\\ \kappa_{I}\left(\gamma_{I}^{\left(2,1\right)}\sigma \left(V^{\left(2\right)}-V_{R}\right)-g_{I}^{\left(1\right)}\right)+\Psi_{g_{I}^{\left(1\right)}}\\ \kappa_{E}\left(\gamma_{E}^{\left(3,1\right)}\sigma \left(V^{\left(3\right)}-V_{R}\right)-g_{E}^{\left(1\right)}\right)+\Psi_{g_{E}^{\left(1\right)}}\\ \frac{1}{C}\left(g_{L}\left(V_{L}-V^{\left(2\right)}\right)+g_{E}^{\left(2\right)}\left(V_{E}-V^{\left(2\right)}\right)+g_{I}^{\left(2\right)}\left(V_{I}-V^{\left(2\right)}\right)\right)+\Psi_{V^{\left(2\right)}}\\ \kappa_{I}\left(\gamma_{I}^{\left(2,2\right)}\sigma \left(V^{\left(2\right)}-V_{R}\right)-g_{I}^{\left(2\right)}\right)+\Psi_{g_{I}^{\left(2\right)}}\\ \kappa_{E}\left(\gamma_{E}^{\left(3,2\right)}\sigma \left(V^{\left(3\right)}-V_{R}\right)-g_{E}^{\left(2\right)}\right)+\Psi_{g_{E}^{\left(2\right)}}\\ \frac{1}{C}\left(g_{L}\left(V_{L}-V^{\left(3\right)}\right)+g_{E}^{\left(3\right)}\left(V_{E}-V^{\left(3\right)}\right)+g_{I}^{\left(3\right)}\left(V_{I}-V^{\left(3\right)}\right)\right)+\Psi_{V^{\left(3\right)}}\\ \kappa_{I}\left(\gamma_{I}^{\left(2,3\right)}\sigma \left(V^{\left(2\right)}-V_{R}\right)-g_{I}^{\left(3\right)}\right)+\Psi_{g_{I}^{\left(3\right)}}\\ \kappa_{E}\left(\gamma_{E}^{\left(1,3\right)}\sigma \left(V^{\left(1\right)}-V_{R}\right)-g_{E}^{\left(3\right)}\right)+\Psi_{g_{E}^{\left(3\right)}} \end{array}\right]$$

$$f\left(\Psi \right)=\;-\frac{1}{\alpha }\Psi$$

$$\Theta =\left[\begin{array}{cc} 0 & 0\\ 0 & \frac{1}{\alpha }Q \end{array}\right]$$

Where **Q** is the covariance matrix of the white noise process driving the colored noise vector **Ψ**, and α is a constant that determines the cutoff frequency of the colored noise.

State-Space model: for this case where the additive noise is colored noise, the model described can be formulated in state-space model of the form:

$$\text{Process Equation}:\;{\dot{x}}^{aug}=f^{aug}\left(x^{aug},I\right)+\Theta \Gamma$$

$$\text{Measurement Equation}:\;z_{k}=h\left(x_{k},k\right)+w_{k}$$

Where $x^{aug}\;\in \;{\mathbb{R}}^{2n}$ is the augmented state vector of the dynamic system at time t, *I* is the exogenous input, $z_{k}\;\in \;{\mathbb{R}}^{d}$ is the measurement at discrete time *t*_*k*_, $f:\;{\mathbb{R}}^{2n}\times \mathbb{R}\to {\mathbb{R}}^{2n}$ is the drift coefficient, $h:\;{\mathbb{R}}^{2n}\times \mathbb{R}\to {\mathbb{R}}^{d}$ is the measurement function, $\Gamma \;\in \;{\mathbb{R}}^{2n}$ and $w_{k}\;\in \;{\mathbb{R}}^{d}$ are vectors of zero mean random Gaussian noise.

As same as the previous model with additive white noise the measurement equation function *h*(*x*_*k*_, *k*) for the colored noise case is dependent on the infra-granular layer membrane potentials (*V*^(3)^).

$$h\left(x_{k},k\right)=V_{k}^{\left(3\right)}$$

Where $V_{k}^{\left(3\right)}$ represents infra-granular membrane potential at discrete time instant *k*.

### Other types of noise processes

In order to examine the effect of Gaussianity assumption for the noise structure in neural model on the performance of Kalman filter in estimating neuronal hidden states, we assumed that the actual continuous system could be driven by different types of noise processes whereas these processes are assumed to be a Wiener process in the Kalman setup.

The system will be examined under the following noise types:

1. Poisson process.
2. Exponential process.
3. Gamma process.
4. Low frequency noise.

The simulation of the neural model driven by Poisson, Exponential and Gamma noise processes is carried out under the assumption that these noise processes are the resultant discrete process after the discretization of the continuous process equation; that is we are assuming that by discretizing the process equation we do not have any prior knowledge about what kind of original continuous noise process could produce these discrete noise processes. Hence, in order to simulate such system driven by these noise types, the continuous system will be discretized with the LL method and the noise process will be added as a discrete process to the discrete dynamics in the same manner that we have simulated the system with additive white noise case. The continuous time dynamics of the system without the noise term is given by:

$$\dot{x}=f\left(x,I\right)$$

The discrete version of this ODE system by the LL method is given by [54]:

$$x_{k+1}\approx x_{k}+J_{k}^{-1}\left[exp\left(J_{k}\Delta t\right)-{Ι}_{e}\right]f\left(x_{k},I\right)$$

Where $J_{k}$ is the Jacobian of ***f*** and Δ*t* is the time interval between samples, and **I**_**e**_ is the identity matrix.

Given this discrete version of the continuous system dynamics, we can add the noise from a discrete process to get a process equation with additive noise as follow:

$$x_{k+1}=x_{k}+J_{k}^{-1}\left[exp\left(J_{k}\Delta t\right)-{Ι}_{e}\right]f\left(x_{k},I\right)+\Omega$$

Where **Ω** represents a discrete Poisson, Exponential or Gamma process.

The state-space representation in discrete time for these cases is:

$$\text{Process Equation}:\;x_{k+1}=x_{k}+J_{k}^{-1}\left[exp\left(J_{k}\Delta t\right)-{Ι}_{e}\right]f\left(x_{k},I\right)+\Omega$$

$$\text{Measurement Equation}:\;z_{k}=h\left(x_{k},k\right)+w_{k}$$

Where $x_{k}\;\in \;{\mathbb{R}}^{n}$ is the state vector of the dynamic system at discrete time *k*, *I* is the exogenous input, $z_{k}\;\in \;{\mathbb{R}}^{d}$ is the measurement at discrete time *t*_*k*_, $f:\;{\mathbb{R}}^{n}\times \mathbb{R}\to {\mathbb{R}}^{n}$ is the drift coefficient, $h:\;{\mathbb{R}}^{n}\times \mathbb{R}\to {\mathbb{R}}^{d}$ is the measurement function, $\Omega \;\in \;{\mathbb{R}}^{n}$ is a discrete noise process, and $w_{k}\;\in \;{\mathbb{R}}^{d}$ is a vector of zero mean random Gaussian noise.

In addition, we will consider the case where the neural model is driven by very slow varying noise considered as filtered white noise. This model will be simulated in the same manner as the colored noise case was simulated but by varying the constant α in the covariance matrix Θ in order to produce noise process having frequency components in the range of 1–5 Hz.

### Hemodynamic model description

In this section, we introduce the mathematical description of the hemodynamic model that relates neural activity (NA) to measured BOLD signals [36, 55, 56]. The model is based on four physiological state variables: vasodilatory signal (*s*), cerebral blood flow (CBF) (*F*), cerebral blood volume (CBV) (*v*), and deoxyhemoglobin content (dHb) (*q*). The hemodynamic model is given by:

$$\dot{s}=u-\kappa s-\lambda \left(F-1\right)$$

$$\dot{F}=s$$

$$\tau \dot{v}=F-v^{1/\beta }$$

$$\tau \dot{q}=\frac{E\left(F,\rho \right)F}{\rho }-\frac{q}{v}v^{1/\beta }$$

The vasodilatory signal (*s*) is a linear function of NA (expressed as firing rate of a given neuronal population) and is subject to auto-regulatory feedback by CBF (*F*). The rate of change in CBV (*v*) is the difference of blood inflow (CBF) and blood outflow (which is function of CBV) from the venous compartment, and the rate of change in dHb (*q*) is the delivered deoxyhemoglobin into the venous compartment minus that expelled (blood outflow (*v*^1/*β*^) times deoxyhemoglobin concentration *q*/*v*). Where *κ* is the rate constant of signal decay, *λ* is the rate constant of feedback regulation, *u* is the input NA, *τ* is the hemodynamic transit time (average time needed for the blood to traverse the venous compartment), *β* is the stiffness or Grubb’s exponent, *E*(*F*, *ρ*) = 1 –(1 − *ρ*)^1/*F*^ is the oxygen extraction function, and *ρ* is the resting oxygen extraction fraction. The hemodynamic model parameters are listed in Table 2.

**Table 2:** Hemodynamic model parameters.

Parameter	Physiologic interpretation	Value	Unit
***κ***	Rate of signal decay	0.65	s^-1^
***λ***	Rate of feedback regulation	0.38	s^-1^
***β***	Grubb’s exponent	0.32	---
***τ***	Hemodynamic transit time	0.98	s
***ρ***	Resting oxygen extraction fraction	0.34	---

The hemodynamic model can be generalized by incorporating an additive noise process, and thus the model can be formulated by the following stochastic differential equations system:

$${\dot{x}}^{HDM}=f^{HDM}\left(x^{HDM},u\right)+\Gamma$$

Where **Γ** is a Gaussian noise vector, the hemodynamic state vector ***x***^*HDM*^ and the model functions ***f***^*HDM*^(.) are:

$$x^{HDM}=\left[\begin{array}{ccc} s & F & \begin{array}{cc} v & q \end{array} \end{array}\right]$$

$$f^{HDM}\left(x^{HDM},u\right)=\left[\begin{array}{c} u-\kappa s-\lambda \left(F-1\right)\\ s\\ \begin{array}{c} \left(F-v^{1/\beta }\right)/\tau \\ \left(\frac{E\left(F,\rho \right)F}{\rho }-\frac{q}{v}v^{1/\beta }\right)/\tau \end{array} \end{array}\right]$$

The observation BOLD signal is a nonlinear function of CBV (*v*), dHb (*q*) and the resting blood volume fraction (*V*0):

$$z^{BOLD}=V_{0}\left[k_{1}\left(1-q\right)+k_{2}\left(1-\frac{q}{v}\right)+k_{3}\left(1-v\right)\right]$$

$$k_{1}=7\rho ,\;k_{2}=2,\;k_{3}=2\rho -0.2$$

It is noted that the state variables CBF (*F*), CBV (*v*), and dHb (*q*) are always positive due to their physiological nature (the flow, volume and deoxyhemoglobin content cannot be negative). Thus, in order to ensure their positive values and the numerical stability of the Kalman filter, their corresponding equations are converted to log space by applying the chain rule after a change of variables, ${\tilde{x}}^{HDM}=ln\;x^{HDM}$ [39].

That is, for any given state variable *x*^*HDM*^ with the state equation $\;{\dot{x}}^{HDM}=f^{HDM}\left(x^{HDM}\right)$:

$${\tilde{x}}^{HDM}=\text{ln}\left(x^{HDM}\right)\Leftrightarrow x^{HDM}=\text{exp}\left({\tilde{x}}^{HDM}\right)$$

$$\Rightarrow \frac{d{\tilde{x}}^{HDM}}{dt}=\frac{d\text{ln}\left(x^{HDM}\right)}{dx^{HDM}}\frac{dx^{HDM}}{dt}=\frac{f^{HDM}\left(x^{HDM}\right)}{x^{HDM}}$$

This transformation will result in the following system:

$$x^{HDM}=\left[\begin{array}{ccc} s & \tilde{F} & \begin{array}{cc} \tilde{v} & \tilde{q} \end{array} \end{array}\right]$$

$$f^{HDM}\left(x^{HDM},u\right)=\left[\begin{array}{c} u-\kappa s-\lambda \left(F-1\right)\\ s/F\\ \begin{array}{c} \left(F-v^{1/\beta }\right)/\tau F\\ \left(\frac{E\left(F,\rho \right)F}{\rho }-\frac{q}{v}v^{1/\beta }\right)/\tau q \end{array} \end{array}\right]$$

When evaluating the BOLD output equation, the log-hemodynamic states are exponentiated, that is $\begin{array}{c} v \end{array}=\text{exp}\left(\tilde{v}\right)$ and $q=\text{exp}\left(\tilde{q}\right)$ are used to compute the predicted observed BOLD signal.

**Fig 3:**
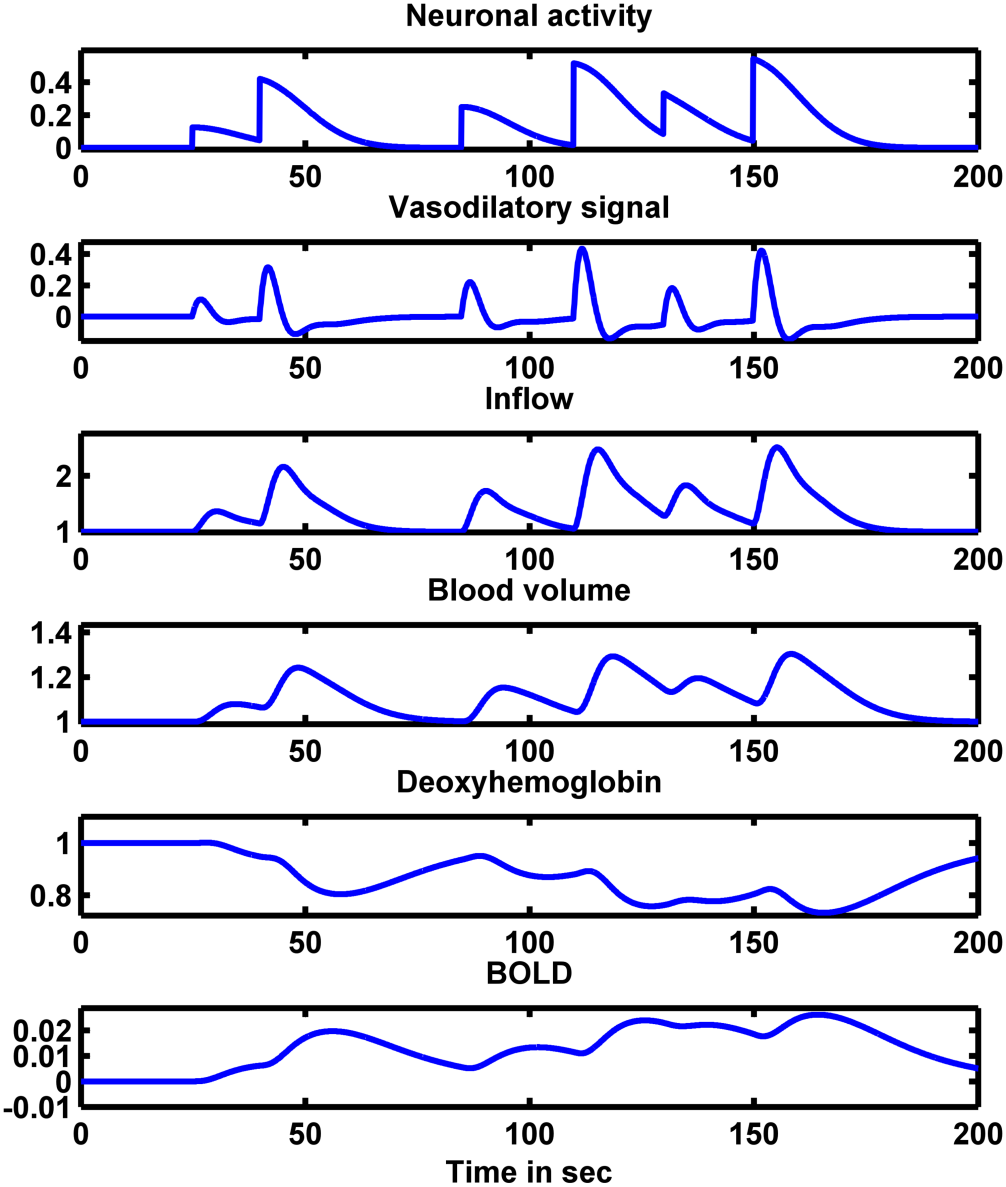
Hemodynamic model. Noiseless BOLD signal (bottom plot) and the dynamics of the hemodynamic variables for a given neural activity (top plot).

Fig 3 shows the behavior of hemodynamic variables for the presented neural activity input (*u*). The model was simulated for 200 *s* with sampling rate *dt* = 0.1 *s* without adding noise to the system.

The hemodynamic model has a smearing effect of the underlying neural activity in a given cortical area; that is the BOLD signal is a low passed filtered version of the underlying neuronal activity. We propose to combine the cortical neuronal model with the hemodynamic model in order to examine the performance of Kalman filter on estimating neuronal states from a noisy smeared observation signal (BOLD signal). Although the neuronal model and the hemodynamic model have different scales of dynamics (neuronal model has dynamics in milliseconds time scale and that of the hemodynamic model is in seconds time scale), we are assuming that the BOLD signal can be observed in milliseconds time scale (in reality the observation BOLD signal has a sampling rate > 1s). This assumption is solely presented to show the general advantage of using CD-CKF in estimating fast dynamics from infrequent observations.

Under this assumption, we propose that the noisy observation BOLD signal is obtained from the infra-granular layer membrane potentials (*V*^(3)^). That is *u* = *σ*(*V*^(3)^) is the input NA and it is a sigmoid function that transforms membrane potential (*V*^(3)^) to firing rate.

The Hybrid Kalman filtering will be tested in two different models that are explained next: (1) a hemodynamic model with the unknown neural activity inputs, and (2) a joint neural-hemodynamic model with known afferent activity to the neural populations.

In the hemodynamic model, the (simulated) observations are assumed to be BOLD signals obtained at a sampling rate (or repeat time TR) of 1 sec and recorded for a period of 64 seconds and the estimation procedure is required to infer neural activity driving the model. This is an input blind deconvolution problem whereby both the nonlinear model parameters and the input are to be delineated from the low frequency observations. The model setup, its constraints and solution methods were chosen to closely resemble those utilized in [32] and are particularly intended to benchmark against the best known performance accuracy of estimation in this field.

In simulating this system, the continuous time dynamics are assumed to be driven by Wiener noise processes. The neural activity took the shape of Gaussian-bump functions with different amplitudes. The IT-1.5 discretization method with a simulation time step of 0.1 *sec* was adopted to generate the continuous observation BOLD signal, which was then artificially resampled at the repeat time (*TR* = 1 *sec*). The states, observation, and inputs to the model are assumed to be driven by random noise having precisions similar to those reported in [32].

The blind deconvolution procedure was applied for two scenarios: (2-a) only the input is unknown, and (2-b) the input as well as two model parameters (rate of signal decay *κ* and rate of feedback regulation *λ*) are unknown.

For the estimation problem, linear interpolations between successive samples were obtained at uniform time steps (*dt* = 0.2, 0.5 *sec*) and are utilized as effective discrete-time observations. Subsequent estimation consisted of a forward pass using the CKF and CD-CKF then a backward smoothing pass (the cubature Rauch–Tung–Striebel smoother, namely CKS and CD-CKS). Since the unknown quantities are the input (first scenario) as well as two parameters (second scenario), the backward pass is necessary to improve on the estimates of the forward pass, as reported in [32]. Furthermore, estimation of unknown parameters (second scenario) was constrained to specific intervals (rate of signal decay *κ* ∈ [0.6–0.9] and rate of feedback regulation *λ* ∈ [0.3–0.5]) and were initialized randomly within these intervals and sampled from uniform distributions.

A total of 100 independent Monte-Carlo simulations were performed and the performance of both CD-CKF and CKF at two sampling rates (*dt* = 0.2, 0.5 *sec*) were assessed (the CD-CKF sampling interval *dt* is divided into *m* steps of length *δ*, where *δ* = *dt*/*m*, and *m* is taken to equal 5).

Finally, for the joint neuronal hemodynamic model, the input to the neural system is assumed known and the sampled BOLD signal is observed. The estimated quantities are the various states of both the hemodynamic and neural sub-models including the neural activity input to the former. The model setup, simulation of the continuous dynamics, and parameters are similar to the ones detailed above.

## Results

In order to compare the performance of CKF and CD-CKF (details of both filter formulations are presented in Appendix), we consider two scenarios of the underlying continuous system and its mathematical representation by the continuous stochastic process equation that is modeled as an SDE driven by a Wiener process:

1. The underlying continuous system is indeed subject to stochastic noise given by a Wiener process.
2. The underlying continuous system is actually subject to colored noise. That is, we are misrepresenting the actual colored noise by assuming that it is a Wiener process.

For both scenarios, we evaluate the performance of CKF and CD-CKF as the accumulative mean square error (MSE) of all normalized states (membrane potentials of granular and supra-granular layers as well as the excitatory and inhibitory conductances of all layers) excluding the observation state (membrane potential of infra-granular layer), over a total of 100 Monte-Carlo simulations.

$$MSE=\frac{1}{M}\sum_{i=1}^{M}E_{i}$$

Where *M* = 8 is the number of unobserved states, and *E*_*i*_ are the elements of a vector *E* defined as:

$$E={\left[\begin{array}{ccccc} E_{V^{\left(1\right)}} & E_{g_{I}^{\left(1\right)}} & E_{g_{E}^{\left(1\right)}} & \begin{array}{ccc} E_{V^{\left(2\right)}} & E_{g_{I}^{\left(2\right)}} & E_{g_{E}^{\left(2\right)}} \end{array} & \begin{array}{cc} E_{g_{I}^{\left(3\right)}} & E_{g_{E}^{\left(3\right)}} \end{array} \end{array}\right]}^{T}$$

For example $E_{V^{\left(1\right)}}$ is defined as follows and all the elements of vector *E* are computed in the same manner:

$$E_{V^{\left(1\right)}}=\frac{1}{NK}\sum_{n=1}^{N}\sum_{k=1}^{K}\frac{{\left({\left(V^{\left(1\right)}\right)}_{k}^{real}-{\left({\hat{V}}^{\left(1\right)}\right)}_{k}^{n}\right)}^{2}}{{\left(V^{\left(1\right)}\right)}_{norm}^{2}}\;N=100$$

Where *K* is the length of the total simulation time vector, ${\left(V^{\left(1\right)}\right)}_{k}^{real}$ is the true state at time *k*, ${\left({\hat{V}}^{\left(1\right)}\right)}_{k}^{n}$ is the estimated state at time *k* in the n^th^ Monte-Carlo run, and ${\left(V^{\left(1\right)}\right)}_{norm}^{2}$ is a normalizing factor.

$${\left(V^{\left(1\right)}\right)}_{norm}^{2}={\left[\text{max }\left({\left(V^{\left(1\right)}\right)}^{real}\right)-\text{min }\left({\left(V^{\left(1\right)}\right)}^{real}\right)\right]}^{2}$$

The normalizing factor is the square of the difference between the maximum and minimum values of a given true state. This factor is introduced in order to make all states magnitude in [0, 1] range.

For each scenario, we generate measurement data from the cortical model with different levels of background noise. We consider a total of eight cases to simulate conditions for a range of different signal-to-noise ratio (SNR) (Table 3). The SNR is defined as:

$$SNR_{dB}=10\;{\text{log}}_{10}\frac{P_{signal}}{P_{noise}}=10{\text{ log}}_{10}\left(\frac{E\left[V_{signal}^{2}\right]}{\sigma_{noise}^{2}}\right)$$

Where *P* is the average power, $E\left[V_{signal}^{2}\right]$ is the mean squared value of signal amplitude, and *σ*_*noise*_ is the standard deviation of the noise.

**Table 3:** SNR values in dB over the observation signal (membrane potential of infra-granular layer).

Case	1	2	3	4	5	6	7	8
SNR of observation signal (dB)	4	7	8	9	11	12	14	18

For each SNR case, we assume that measurement data is collected at different sampling time steps (*dt*), with *dt* = 0.1, 0.5, 1, 2, 4, 8 *ms*, in order to examine the effect of decreasing the sampling rate on the estimation error.

The measurement update for the observations in both filtering cases is obviously dictated by the assumed sampling rate for the CKF, the time update occurs in concurrence with the measurements every *dt* millisecond. For the CD-CKF, however, the time update occurs every *δ* milliseconds where each sampling interval *dt* is divided into *m* steps of length *δ*, where *δ* = *dt*/*m* (where *m* is taken to equal 5).

We study the performance of both filters for different noise scenarios (7 cases), several sampling rates, (6 cases) and signal-to-noise ratios (8 cases). We here consider in detail two main noise scenarios (white and colored noise).

### First scenario: White noise

We assume here that the continuous time system is driven by a Wiener noise process for different SNRs. For the purpose of simulation, we adopt a time step of 0.01 *ms* in IT-1.5 discretization method in order to generate measurement data [57]. We subsequently artificially resample the output data of the simulation at different sampling rates (*dt* = 0.1, 0.5, 1, 2, 4, 8 *ms*) and make the resampled data available as measurement data for the two filters.

**Fig 4:**
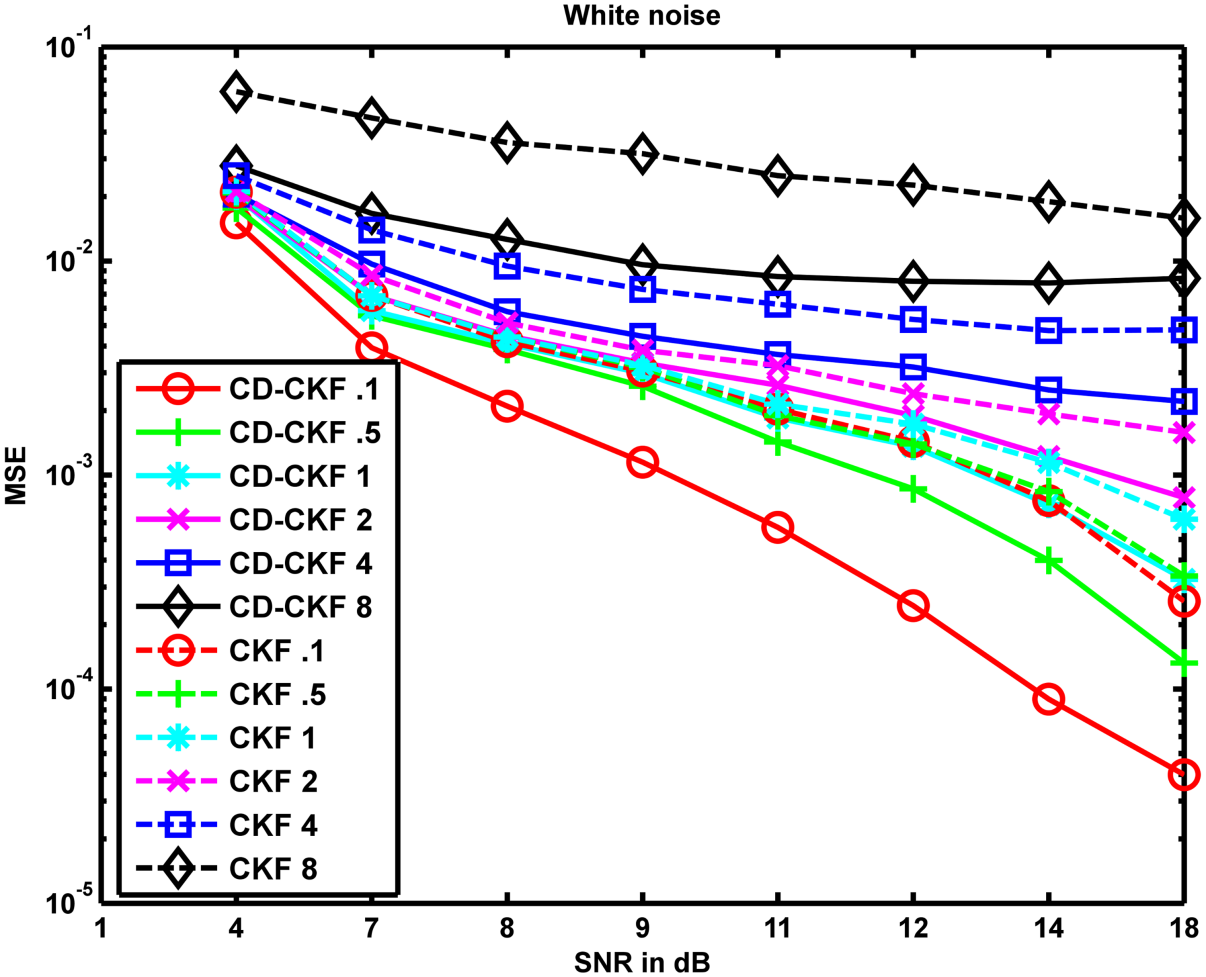
Performance of CD-CKF and CKF under white noise. MSE values averaged over 100 Monte-Carlo runs of CD-CKF and CKF for different SNRs and different sampling rates where the underlying system is perturbed by additive white noise.

Fig 4 shows the MSE values averaged over 100 Monte-Carlo runs of CD-CKF and CKF for different SNRs at different sampling rates. From the figure, it is clear that the CD-CKF outperformed the CKF for all cases. For a given SNR, both filters showed improved convergence to true underlying processes as the sampling rate decreases. However, for a given sampling rate, the CD-CKF scored smaller MSE values than those of the CKF.

**Fig 5:**
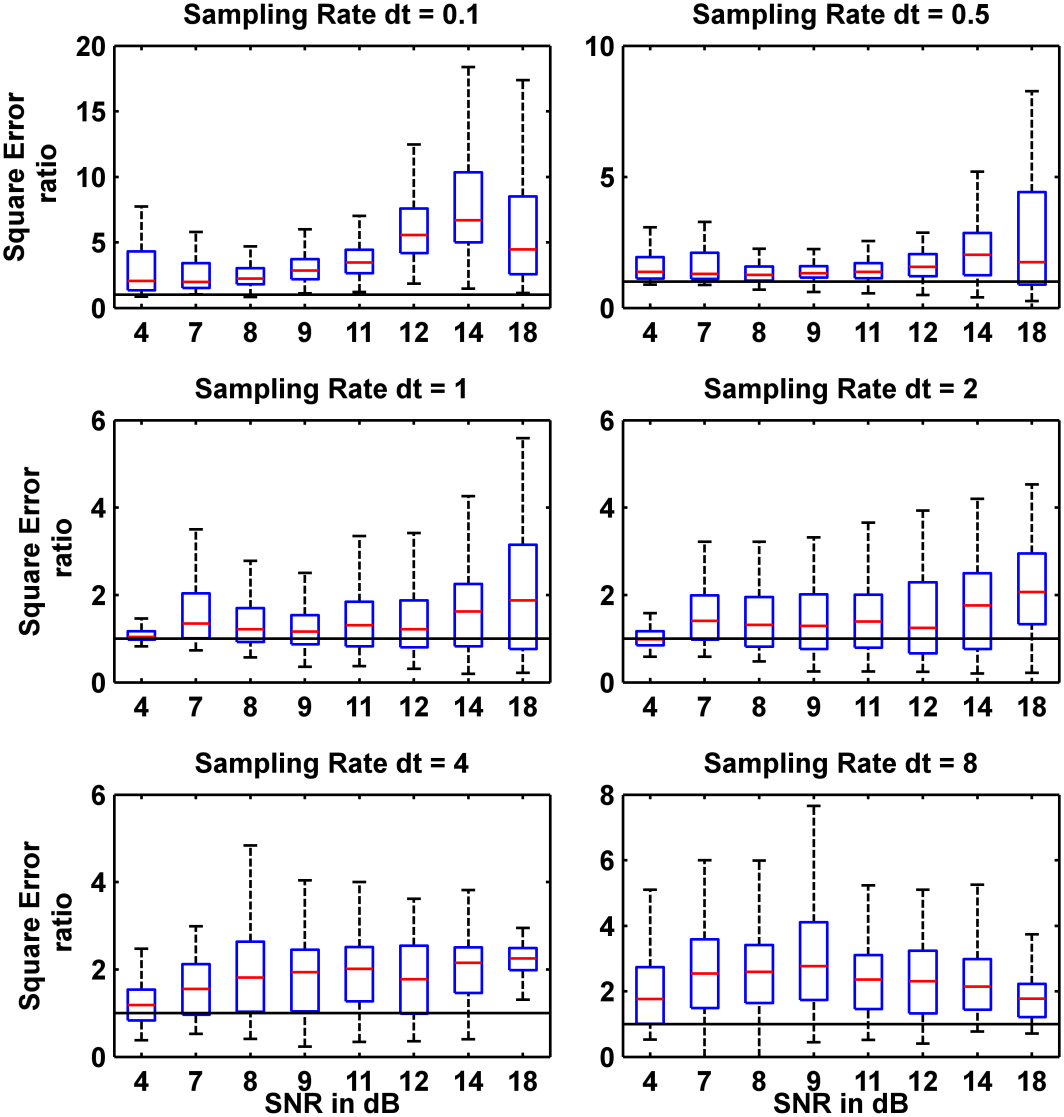
Distribution of squared error ratios of 100 Monte-Carlo runs for different sampling rates and different SNRs for the additive white noise case. Each box plot is based on 100 data samples. The horizontal red lines inside the boxes are the medians, The boxes contain 50% of the samples where the lower and upper edges of each box are the 25^th^ and 75^th^ percentiles, and the "whiskers" above and below the box indicate the range of the samples (the locations of minimum and maximum sample data points that are not considered outliers).

In order to statistically examine how better the CD-CKF performed than the CKF, Fig 5 shows the boxplots at different sampling rates of CD-CKF squared error to CKF squared error ratio. Each boxplot refers to ratios computed for a given SNR at a given sampling rate for the 100 Monte-Carlo runs. The squared error ratio was computed as follow:

$$R^{n}=\frac{{\left(SE^{n}\right)}^{CD-CKF}}{{\left(SE^{n}\right)}^{CKF}}\;n=1,2,\ldots ,100$$

Where the squared error of each filter is defined as:

$${\left(SE^{n}\right)}^{f}=\frac{1}{M}\sum_{i=1}^{M}IE_{i}^{n}$$

Where *f* stands for the filter type, *n* is the n^th^ Monte-Carlo run and *i* represents the state with *M* = 8, and $IE_{i}^{n}$ are the elements of a vector *IE*^*n*^ defined as:

$$IE^{n}={\left[\begin{array}{ccccc} IE_{V^{\left(1\right)}}^{n} & IE_{g_{I}^{\left(1\right)}}^{n} & IE_{g_{E}^{\left(1\right)}}^{n} & \begin{array}{ccc} IE_{V^{\left(2\right)}}^{n} & IE_{g_{I}^{\left(2\right)}}^{n} & IE_{g_{E}^{\left(2\right)}}^{n} \end{array} & \begin{array}{cc} IE_{g_{I}^{\left(3\right)}}^{n} & IE_{g_{E}^{\left(3\right)}}^{n} \end{array} \end{array}\right]}^{T}$$

For example $IE_{V^{\left(1\right)}}^{n}$ is defined as follows and all remaining elements are computed in the same manner:

$$IE_{V^{\left(1\right)}}^{n}=\frac{1}{K}\sum_{k=1}^{K}\frac{{\left({\left(V^{\left(1\right)}\right)}_{k}^{real}-{\left({\hat{V}}^{\left(1\right)}\right)}_{k}^{n}\right)}^{2}}{{\left(V^{\left(1\right)}\right)}_{norm}^{2}}$$

Where *K* is the length of the total simulation time vector, ${\left(V^{\left(1\right)}\right)}_{k}^{real}$ is the true state at time *k*, ${\left({\hat{V}}^{\left(1\right)}\right)}_{k}^{n}$ is the estimated state at time *k* in the n^th^ Monte-Carlo run, and ${\left(V^{\left(1\right)}\right)}_{norm}^{2}$ is a normalizing factor.

$${\left(V^{\left(1\right)}\right)}_{norm}^{2}={\left[\text{max }\left({\left(V^{\left(1\right)}\right)}^{real}\right)-\text{min }\left({\left(V^{\left(1\right)}\right)}^{real}\right)\right]}^{2}$$

Where *f* stands for the filter type, *n* is the n^th^ Monte-Carlo run and *i* represents the state with *M* = 8, and *K* is the length of the total simulation time vector.

As seen in Fig 5, the median ratio was consistently greater than one for all simulations. Seen in terms of the length of sampling time step *dt*, it is noted that the CD-CKF to CKF ratio is highest for very small time step (*dt* = 0.1 *ms*) and large time steps (*dt* = 4 − 8 *ms*). At intermediate time step, (*dt* = 1 − 2 *ms*), the ratio is nearly unity (performance is comparable) only for very low SNR (4 dB). In terms of the SNR variation, it is noted that the ratio was the highest for all SNR levels at very small time steps (*dt* = 0.1 *ms*, or frequent measurments). An increase in the SNR value generally improves the ratio for smaller time steps (both in median value and overall spread but not at wider time steps (*dt* = 4 − 8 *ms*), where the ratio does not increase in neither median nor overall spread with large SNR).

### Second scenario: Colored noise

In this case, we consider the continuous time system when subjected to a colored noise process (which violates the assumptions taken in both finding the CD-CKF and CKF estimates).

$$\dot{x}\left(t\right)=f\left(x,I\right)+\Psi$$

Where **Ψ** is filtered white noise.

**Fig 6:**
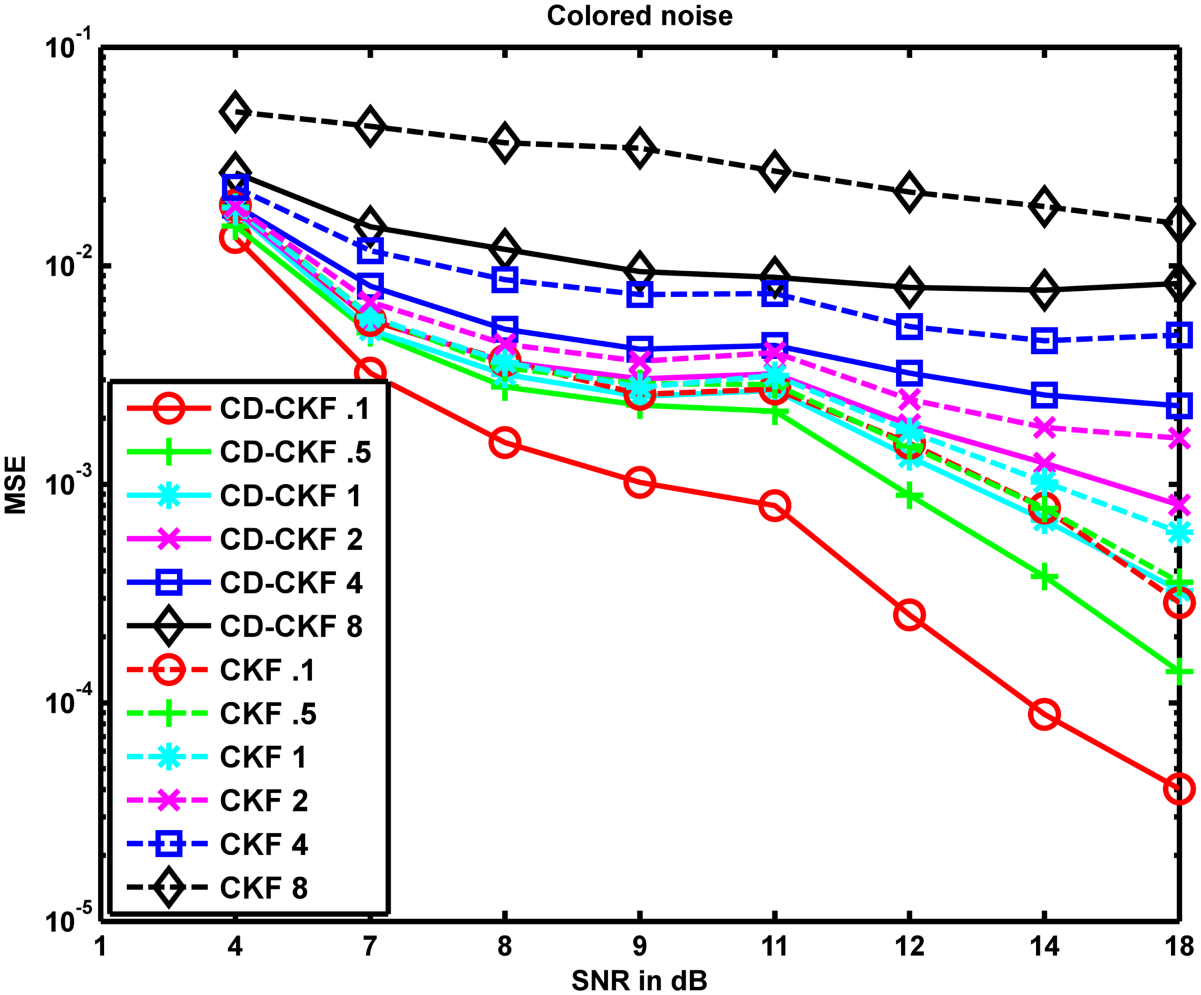
Performance of CD-CKF and CKF under colored noise. MSE values averaged over 100 Monte-Carlo runs of CD-CKF and CKF for different SNRs and different sampling rates where the underlying system is perturbed by additive colored noise.

We again simulate the system using the IT-1.5 (as described in section "Other types of noise processes" below) with *dt* = 0.1 *ms* to produce observations which then were resampled at different sampling rates for the application of filters and the MSE values averaged over 100 Monte-Carlo runs for both filters under different SNRs and sampling rates were computed and shown in Fig 6.

**Fig 7:**
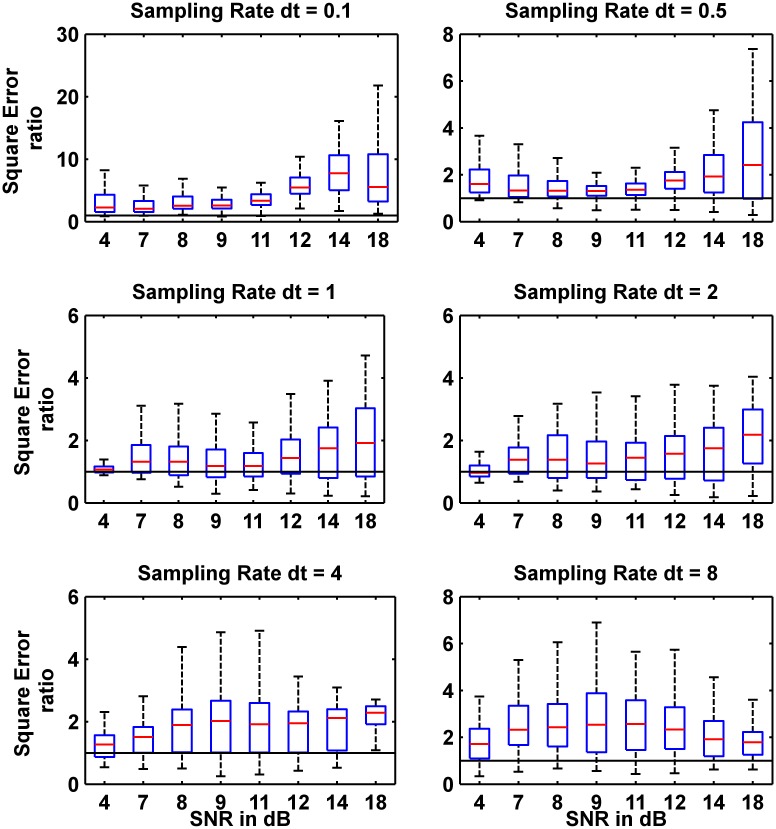
Distribution of squared error ratios of 100 Monte-Carlo runs for different sampling rates and different SNRs for the additive colored noise case.

It is noted here that these results are in line with those observed in the first scenario; the CD-CKF performed better than the CKF with an improvement of performance with decreasing sampling times for both filters. Fig 7 shows the boxplots of squared error ratio for different SNRs and sampling times for the 100 Monte-Carlo runs. When compared to the white noise case, dependence of the ratio on time steps and SNR in the colored noise case follows a similar pattern (Figs 5 and 7): The ratio is highest at the two opposite ends of the sampling rate (*dt* = 0.1 *ms* and *dt* = 8 *ms*), and the performance improves with increasing SNR particularly at intermediate step lengths (*dt* = 1 − 2 *ms*) but not at large time steps (*dt* = 8 *ms*). Notably, however, the actual value of the ratio was higher (CD-CKF was better) for low SNR in the colored noise case of all sampling intervals. That is, it is apparent that the CD-CKF is more resilient to additive colored-noise particularly under large noise components.

### Effect of the simulation method

The continuous time simulations of the system were performed using IT-1.5 discretization methods. Therefore, and since the CD-CKF uses the IT-1.5 method to discretize the continuous process equation while the CKF uses the local linearization scheme (LL), a legitimate concern is whether the improvement in results obtained with CD-CKF is simply caused by the matching discretization techniques in CD-CKF and system simulation.

**Fig 8:**
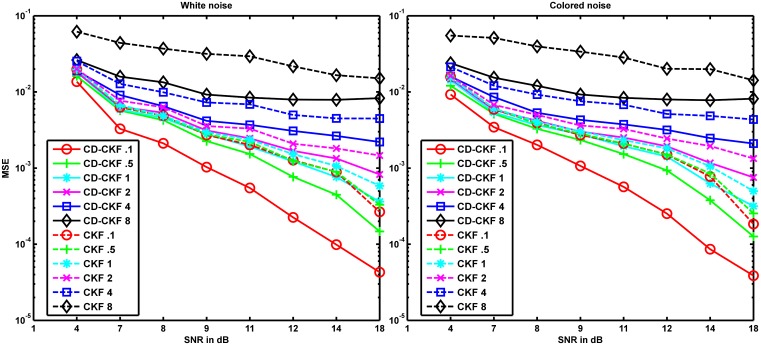
Performance of CD-CKF and CKF with the observations obtained by simulating the system using the LL discretization method. MSE of CD-CKF and CKF for different SNRs and different sampling rates where the underlying system is perturbed by additive white noise (left) and colored noise (right).

We therefore repeated the estimation problem for both scenarios with the observations obtained by simulating the system using the LL discretization method. The MSE values averaged over 100 Monte-Carlo runs of both filters for additive white and colored noise in this case are shown in Fig 8. We can see that CD-CKF still performed better than the CKF and hence the simulation method has no effect on the relative performance of both filters.

### Other types of noise processes

In the Kalman estimation framework, the additive noise is assumed to be derived from a Wiener process. To address the sensitivity of the obtained results on this assumption, we examine in this section the performance of CD-CKF and CKF under the assumption that the actual continuous time system is driven by other forms of additive noise processes. Here, the continuous system is discretized with the LL method and the noise process is added as a discrete process to the discrete dynamics in the same manner that we have simulated the system with additive white noise case. (Note here that the correspondence of discrete white noise to a continuous Wiener process is a well-known phenomenon. The discrete non-white processes as incorporated here, however, are assumed to correspond to other continuous processes that are generally unknown and are intended solely to study the robustness of the Kalman filtering techniques). In particular, the performance of the two filters is examined under DT noise derived from (i) Poisson, (ii) Exponential, and (iii) Gamma distributions.

A final noise case to be considered is that of (iv) additive very slowly varying noise that is concentrated in the frequency range of the observed signals. Specifically, noise is modeled as filtered white noise having frequency components of 1–5 Hz which is in the frequency range of the measured membrane potential.

#### Measuring the accuracy of the estimates

In order to examine the performance of both filters for the above noise scenarios, we conducted a deviation analysis to examine the inaccuracy rates of each filter. The filer is said to be inaccurate when the normalized error between estimated and real states exceeds 20%. For the deviation analysis, we will introduce two types of metrics that will be used as a measure of the inaccuracy of a given filter.

1. A probability of inaccuracy measure *PI*:
  *PI* is regarded as the probability of the obtaining an inaccurate state estimate, that is, the estimated states being 20% far from the true states. It can further be considered as the total fraction of time when the estimated states were 20% far away from the true states.
  $$PI=\frac{1}{M}\sum_{i=1}^{M}P_{i}$$
  Where *M* = 8 is the number of unobserved states, and *P*_*i*_ are the elements of a vector *P* defined as:
  $$P={\left[\begin{array}{cccccc} P_{V^{\left(1\right)}} & P_{g_{I}^{\left(1\right)}} & P_{g_{E}^{\left(1\right)}} & P_{V^{\left(2\right)}} & \begin{array}{cc} P_{g_{I}^{\left(2\right)}} & P_{g_{E}^{\left(2\right)}} \end{array} & \begin{array}{cc} P_{g_{I}^{\left(3\right)}} & P_{g_{E}^{\left(3\right)}} \end{array} \end{array}\right]}^{T}$$
  where *P*_*x*_, $x\in \left\{V^{\left(1\right)},\;g_{I}^{\left(1\right)},\;g_{E}^{\left(1\right)},\;V^{\left(2\right)},g_{I}^{\left(2\right)},\;g_{E}^{2},\;g_{I}^{\left(3\right)},\;g_{E}^{\left(3\right)}\right\}$ is defined as follows
  $$P_{x}=\frac{1}{NK}\sum_{n=1}^{N}\sum_{k=1}^{K}U\left[{\left(\frac{x\left[k\right]-{\hat{x}}_{n}\left[k\right]}{x\left[k\right]}\right)}^{2}>\theta^{2}\right]$$
  Where *K* is the length of the total simulation time vector, N is the total number of Monte-Carlo simulations (N = 100), *x*[*k*] is the true states at time *k*, ${\hat{x}}_{n}\left[k\right]$ is the estimate of the state *x* at time *k* in the n^th^ Monte-Carlo run, *θ* = 0.2 is the accuracy threshold, and *U* is the Heaviside function
  $$U\left[z^{2}>\theta^{2}\right]=\{\begin{array}{cc} 0 & if\;z^{2}<\theta^{2}\\ 1 & if\;z^{2}\geq \theta^{2} \end{array}$$
2. A level of inaccuracy measure (LI):
  This is intended to quantify the amount of inaccuracy rather than just computing its probability. *LI* is computed as the total area under the curve where the estimated states were 20% far from the true states.
  $$LI=\frac{1}{M}\sum_{i=1}^{M}A_{i}$$
  Where *M* = 8 is the number of unobserved states, and *A*_*i*_ are the elements of a vector *A* defined as:
  $$A={\left[\begin{array}{cccccc} A_{V^{\left(1\right)}} & A_{g_{I}^{\left(1\right)}} & A_{g_{E}^{\left(1\right)}} & A_{V^{\left(2\right)}} & \begin{array}{cc} A_{g_{I}^{\left(2\right)}} & A_{g_{E}^{\left(2\right)}} \end{array} & \begin{array}{cc} A_{g_{I}^{\left(3\right)}} & A_{g_{E}^{\left(3\right)}} \end{array} \end{array}\right]}^{T}$$
  Where *A*_*x*_, $x\in \left\{V^{\left(1\right)},\;g_{I}^{\left(1\right)},\;g_{E}^{\left(1\right)},\;V^{\left(2\right)},g_{I}^{\left(2\right)},\;g_{E}^{2},\;g_{I}^{\left(3\right)},\;g_{E}^{\left(3\right)}\right\}$ is defined as
  $$A_{x}=\frac{1}{NK}\sum_{n=1}^{N}\sum_{k=1}^{K}{\left(\frac{x\left[k\right]-{\hat{x}}_{n}\left[k\right]}{\Delta x\left[k\right]}\right)}^{2}\left(U\left[{\left(\frac{x\left[k\right]-{\hat{x}}_{n}\left[k\right]}{x\left[k\right]}\right)}^{2}>\theta^{2}\right]\right)$$
  Where *K* is the length of the total simulation time vector, N is the total number of Monte-Carlo simulations (N = 100), *x*[*k*] is the true state at time *k*, ${\hat{x}}_{n}\left[k\right]$ is the estimate of the state *x* at time *k* in the n^th^ Monte-Carlo run, and Δ*x*[*k*] is a normalizing range factor.
  Δx[k]=max(x[k])−min(x[k])

The filtering performances is evaluated using the above two measures (PI, LI) under an additive white noise assumption as a baseline case and subsequently under the other noise scenarios for comparison.

#### Performance of the CD-CKF

The PI and LI measures of the CD-CKF filter for white noise case are listed in Tables 4 and 5, respectively. The values are the percentage out of 100 Monte-Carlo runs of the time where the estimated states were 20% away from the true states. From Tables 4 and 5, we note that the total fraction of time (PI) as well as the level of deviation from being within 20% from the true states (LI) are both decreasing with increasing SNR. That is, the states will wander off for shorter periods of time from the true value and the amount of deviation (error) will be less as the SNR is increased. In terms of the sampling time *dt*, and for a given SNR, these measures are decreasing with *dt*, that is, the CD-CKF will produce more accurate estimates more often (longer periods of time) with smaller sampling rates.

**Table 4:** Probability rates of CD-CKF filter for white noise. Each number denotes the percentage out of 100 Monte-Carlo simulations of the time where the estimated states were 20% away from the true states.

		CD-CKF Probability measure PI: white noise case					
		*dt* in *ms*					
		0.1	0.5	1	2	4	8
SNR in dB	4	6.67	7.69	7.75	7.53	8.06	11.61
	7	2.37	3.33	3.51	3.77	4.79	6.64
	8	1.37	2.43	2.52	2.84	3.69	4.94
	9	0.68	1.76	2.00	2.28	3.23	4.03
	11	0.19	0.92	1.18	1.86	2.79	3.68
	12	0.003	0.46	0.75	1.28	2.45	3.47
	14	0	0.08	0.23	0.78	1.87	3.37
	18	0	0	0	0.45	1.63	3.26

**Table 5:** Area rates of CD-CKF filter for white noise. Each number denotes the percentage out of 100 Monte-Carlo simulations of the area under the curve where the estimated states were 20% away from the true states.

		CD-CKF Area measure LI: white noise case					
		*dt* in *ms*					
		0.1	0.5	1	2	4	8
SNR in dB	4	2.86	3.22	3.24	3.05	3.06	4.38
	7	0.80	1.18	1.23	1.33	1.62	2.56
	8	0.41	0.81	0.85	0.93	1.17	1.95
	9	0.18	0.54	0.62	0.69	0.98	1.66
	11	0.05	0.26	0.34	0.54	0.85	1.54
	12	0.001	0.12	0.21	0.34	0.74	1.47
	14	0	0.02	0.06	0.19	0.56	1.45
	18	0	0	0	0.11	0.49	1.49

**Fig 9:**
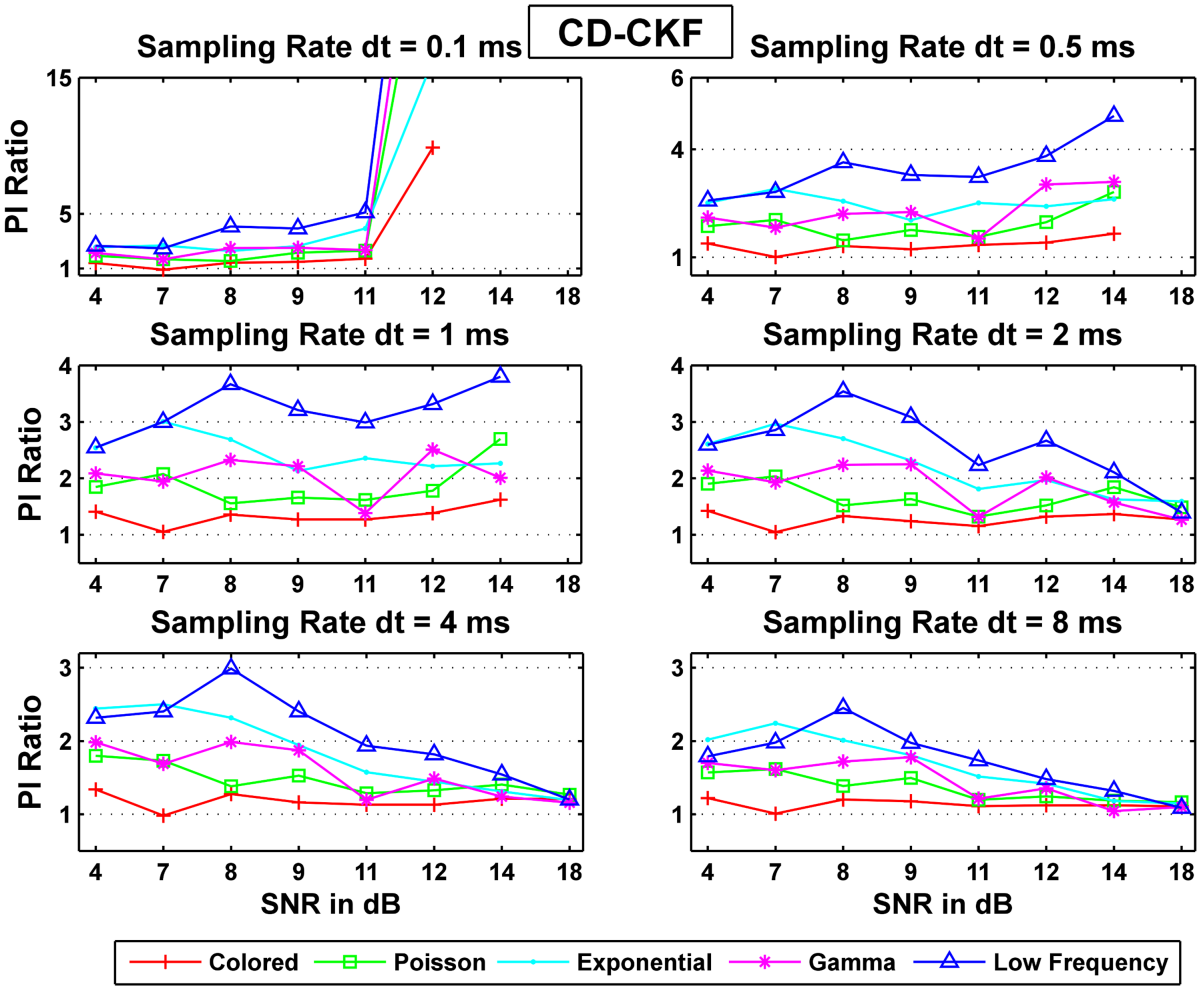
Probability ratios *PI*_*noise*_/*PI*_*white*_ for CD-CKF for different sampling intervals and noise structures.

We also evaluated the CD-CKF performance when the whiteness assumption is violated. Fig 9 summarizes the deterioration in performance using the error ratio *PI*_*noise*_/*PI*_*white*_ computed for different time steps and multiple SNRs. We note that this ratio was closest to unity for additive DT colored noise while it was largest for additive low frequency noise.

Importantly, Poisson-type noise, a common approximation of background input in neuronal networks, showed mild performance deviation from that of white noise. At small time steps (*dt* = 0.1–1 *ms*), the computed ratio is (very) high since the CD-CKF white noise performance was significantly better than the CD-CKF performance under other noise types. This distinction becomes less obvious for larger time steps (*dt* ≥ 2*ms*), particularly with increasing SNR. Hence the CD-CKF performance was most sensitive to whiteness assumption for small time steps and is least sensitive to this assumption at large time steps and high SNR values.

#### Performance of the CKF

**Fig 10:**
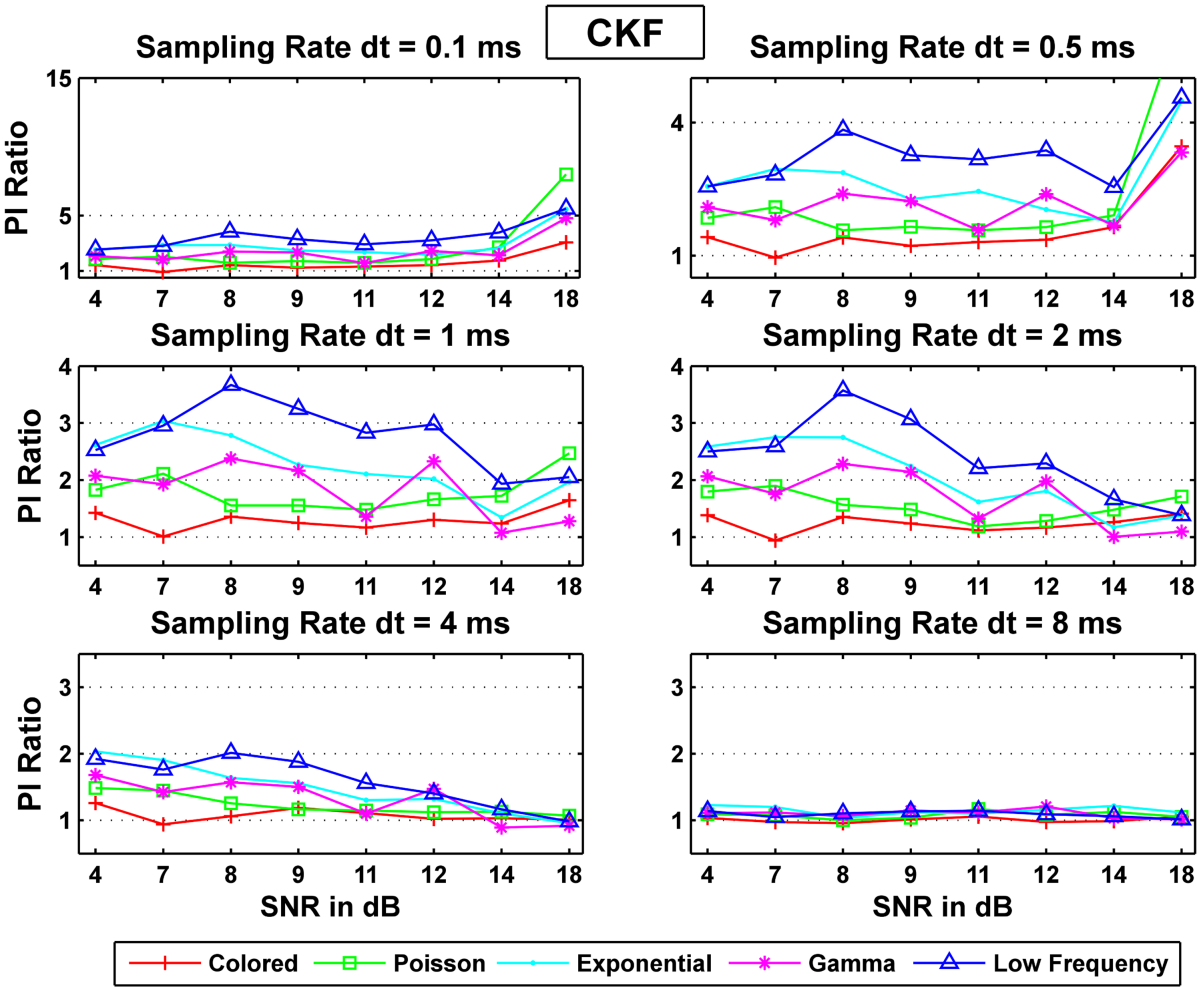
Probability ratios *PI*_*noise*_/*PI*_*white*_ for the CKF estimation.

We here conducted an analysis of the CKF performance for different additive noise cases and summarize the results in Tables 6 and 7, and Fig 10. In Fig 10, it is again seen that colored noise had the closest performance to white while low frequency noise was the farthest from satisfying the whiteness assumption. Importantly, Fig 10 show that quality of the CKF estimates mildly deteriorate from white noise to other noise types as the SNR increase at low sampling rates (*dt* = 0.1 *ms*), unlike the performance sensitivity shown for the CD-CKF at small time. Furthermore, the CKF performance becomes largely independent of the noise structure at large sampling rates (*dt* = 8*ms*).

**Table 6:** Probability rates of CKF filter for white noise. Each number denotes the percentage out of 100 Monte-Carlo simulations of the time where the estimated states were 20% away from the true states.

		CKF Probability measure PI: white noise case					
		*dt* in *ms*					
		0.1	0.5	1	2	4	8
SNR in dB	4	7.98	7.82	7.74	7.76	10.8	24.49
	7	3.77	3.71	3.57	3.95	7.06	21.49
	8	2.63	2.62	2.59	2.68	5.29	17.63
	9	2.1	2.16	2.15	2.29	4.67	14.72
	11	1.35	1.28	1.43	1.94	4.23	12.66
	12	0.9	0.9	1.01	1.34	3.46	11.61
	14	0.35	0.44	0.64	1.03	3.27	10.54
	18	0.02	0.04	0.17	0.67	3.43	9.28

**Table 7:** Area rates of CKF filter for white noise. Each number denotes the percentage out of 100 Monte-Carlo simulations of the area under the curve where the estimated states were 20% away from the true states.

		CKF Area measure LI: white noise case					
		*dt* in *ms*					
		0.1	0.5	1	2	4	8
SNR in dB	4	3.24	3.21	3.26	3.22	3.99	9.72
	7	1.33	1.31	1.25	1.42	2.4	8.42
	8	0.87	0.88	0.86	0.88	1.67	6.77
	9	0.65	0.67	0.66	0.7	1.42	5.63
	11	0.4	0.37	0.41	0.59	1.26	4.7
	12	0.25	0.25	0.29	0.38	1.01	4.33
	14	0.09	0.11	0.17	0.27	0.93	3.94
	18	0.005	0.01	0.04	0.17	0.97	3.48

#### Comparison of CD-CKF and CKF

To arrive at a simple and concise comparative assessment of the performance of CD-CKF and CKF for different noise types, we plot the probability and area rates for each scenario (after being normalized by the worst probability *PI*_*o*_ and area performance *LI*_*o*_ values respectively) of the CD-CKF with white noise case (which were obtained for lowest SNR = 4 dB and largest time step *dt* = 8 ms).

**Fig 11:**
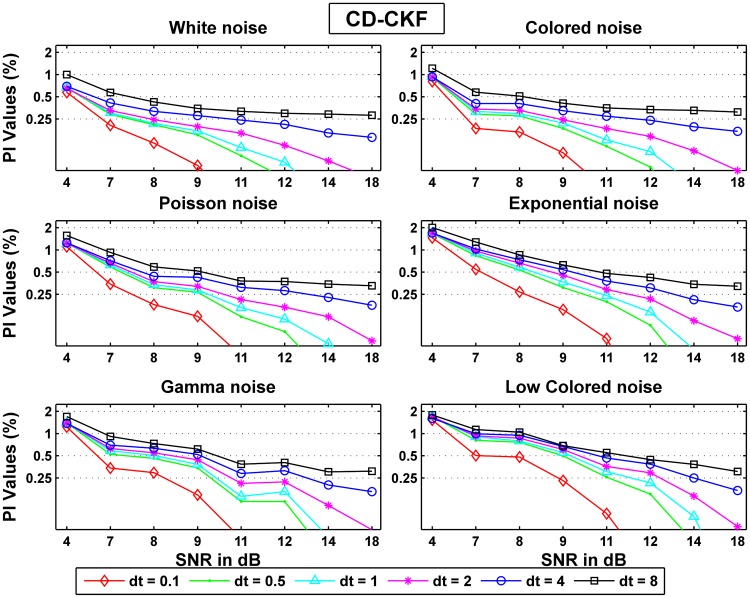
Normalized probability rates for the CD-CKF.

**Fig 12:**
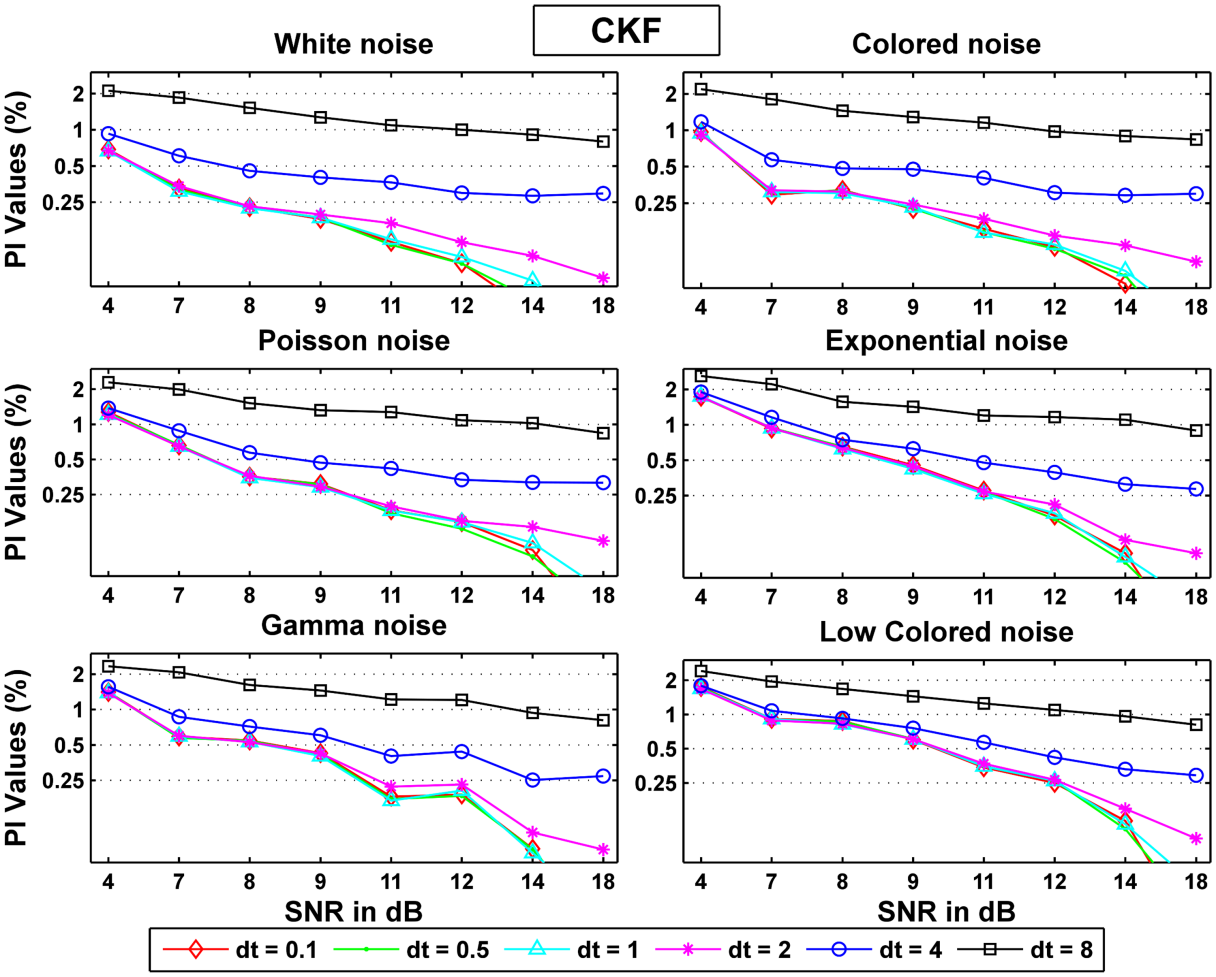
Normalized probability rates for the CKF.

Figs 11 and 12 show the normalized probability rates for the CD-CKF and the CKF respectively. Here again, we notice that while both filters improve their performance as the SNR increase, the CD-CKF has a sharper SNR-related improvement (faster slop decline) for a given time step and all noise types tested. Furthermore, the CD-CKF performance improved steadily with smaller time steps while the CKF performance remains essentially unchanged as the sampling time steps decrease below *dt* = 1 ms.

**Fig 13:**
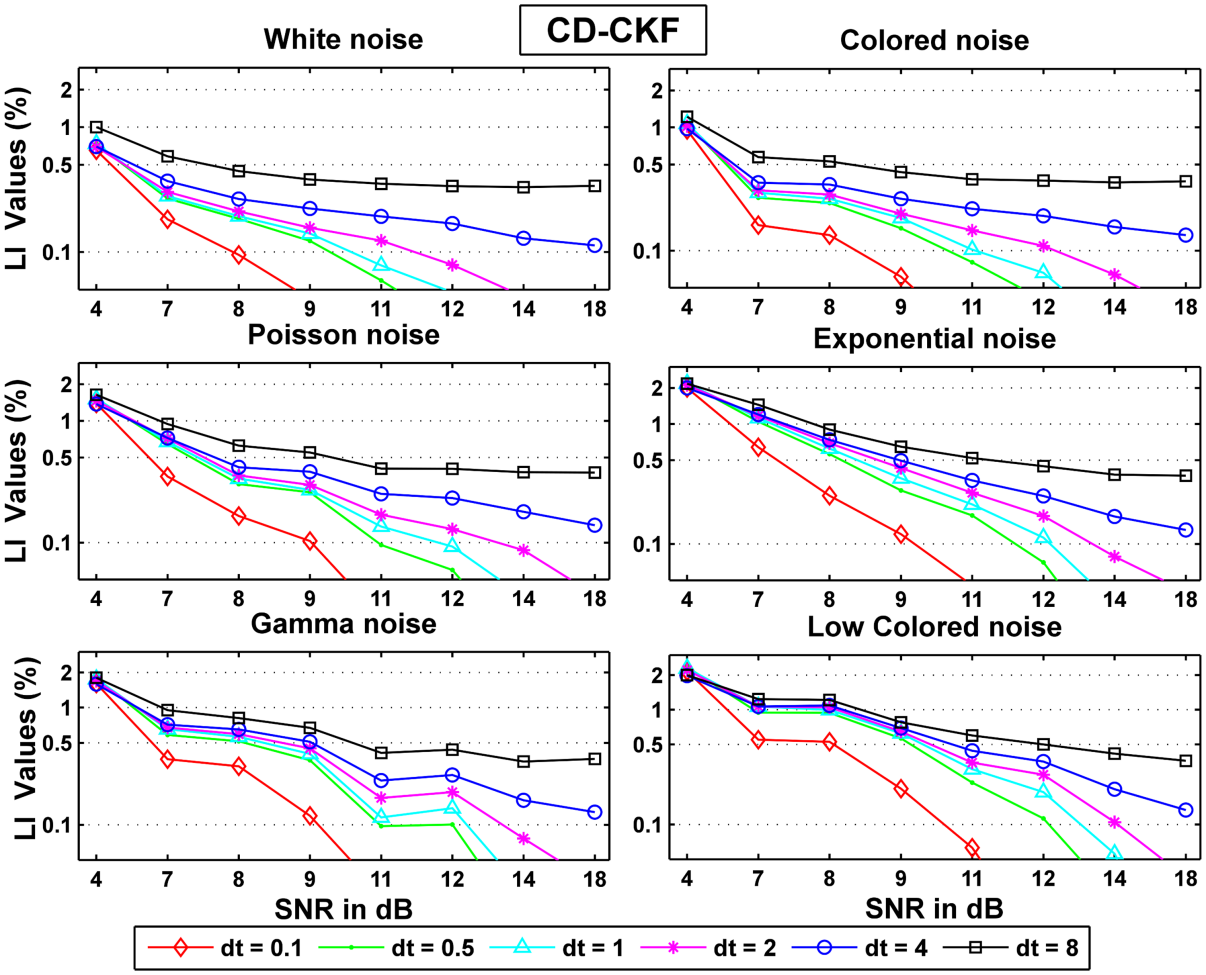
Normalized area rates for the CD-CKF.

**Fig 14:**
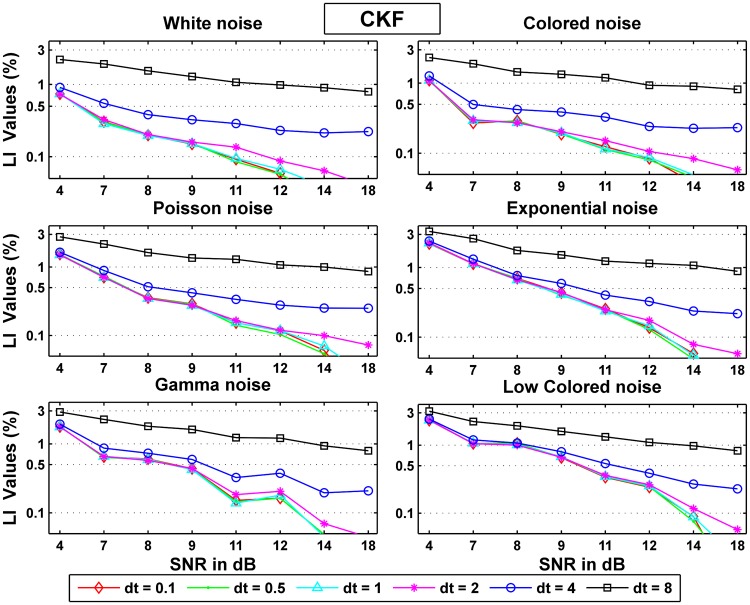
Normalized area rates for the CKF.

Figs 13 and 14 show the normalized area rates for the CD-CKF and the CKF respectively.

To examine the performance improvement of the CD-CKF over the CKF, we computed the ratios of the values obtained for CKF over those of CD-CKF for both the probability measure PI and the inaccuracy measure LI, that is

$$Ratio_{i,j,l}={\left(\frac{P_{i,j}^{CKF}}{P_{i,j}^{CD-CKF}}\right)}_{l}$$

**Fig 15:**
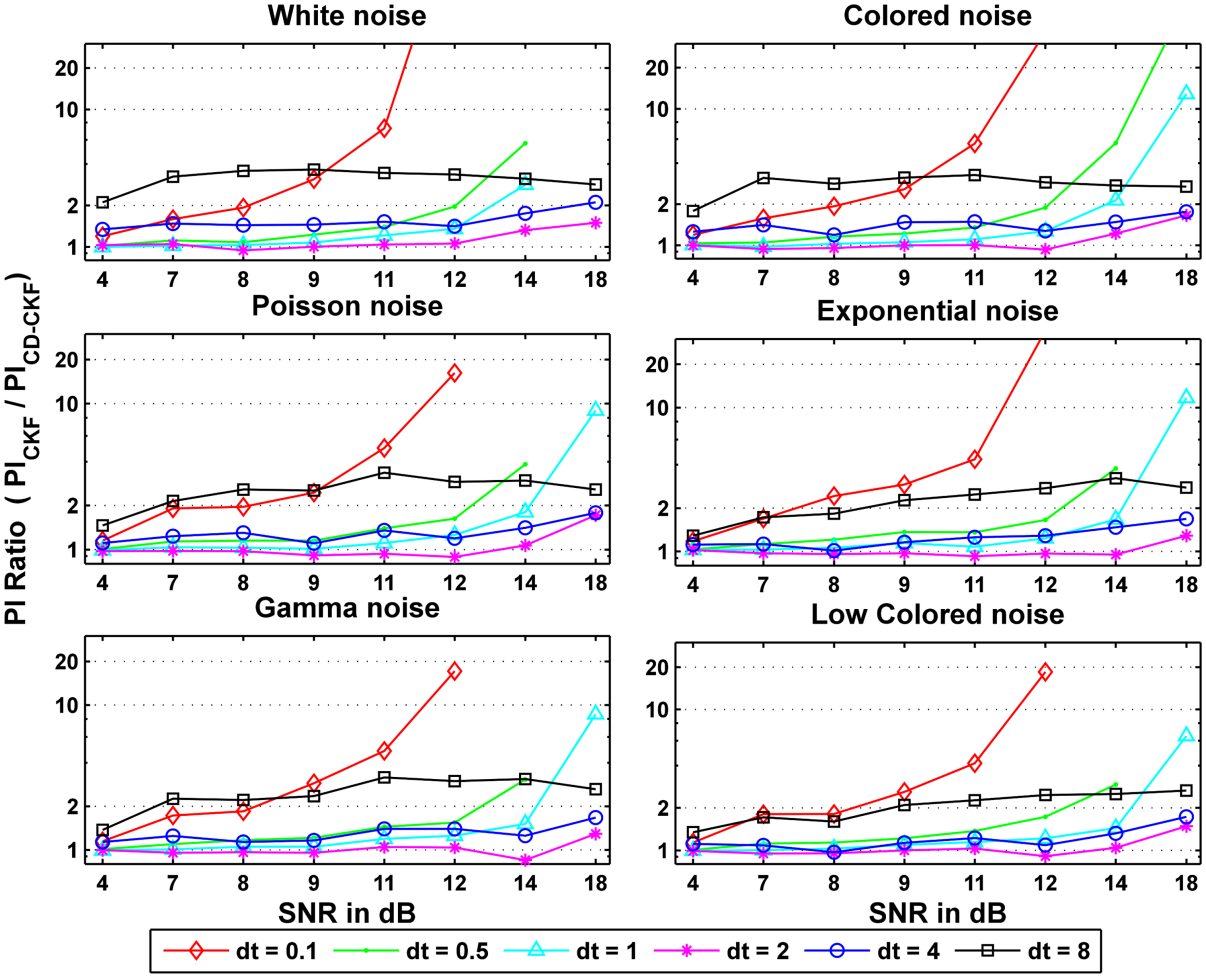
Ratios of the performance index PI of the CKF to that the CD-CKF values for different process noise structures and observation noise levels.

Where *l* denotes the noise type, *i* for SNR value, and *j* denotes the sampling rate *dt* value. Fig 15 shows the ratio of the probability of the CKF values to that of the CD-CKF values. We can see that the CD-CKF quality of estimates are better (ratio >1) for most cases tested.

First, for large sampling intervals (*dt* = 8*ms*), the CD-CKF is nearly twice more accurate for Gaussian noise (white, colored) with the CKF performance improving as the SNR increases (slope of ratio decreases). The CD-CKF is also significantly more accurate under other noise types (Poisson, Exponential, low frequency) with the CKF performance lagging behind that of the CKF as the SNR increases (slope of ratio increases).

Second, for small sampling intervals (*dt* = 0.1, 0.5 and 1*ms*), the CD-CKF performance improves at a much higher rate compared to that of the CKF as the SNR increases, regardless of the noise structure assumed.

Finally, for intermediate sampling step (*dt* = 2–4*ms*), the CD-CKF performance is comparable to that of the CKF for a wide SNR range and is only significantly better for the largest SNR tested (18 dB)

### Hemodynamic model

**Fig 16:**
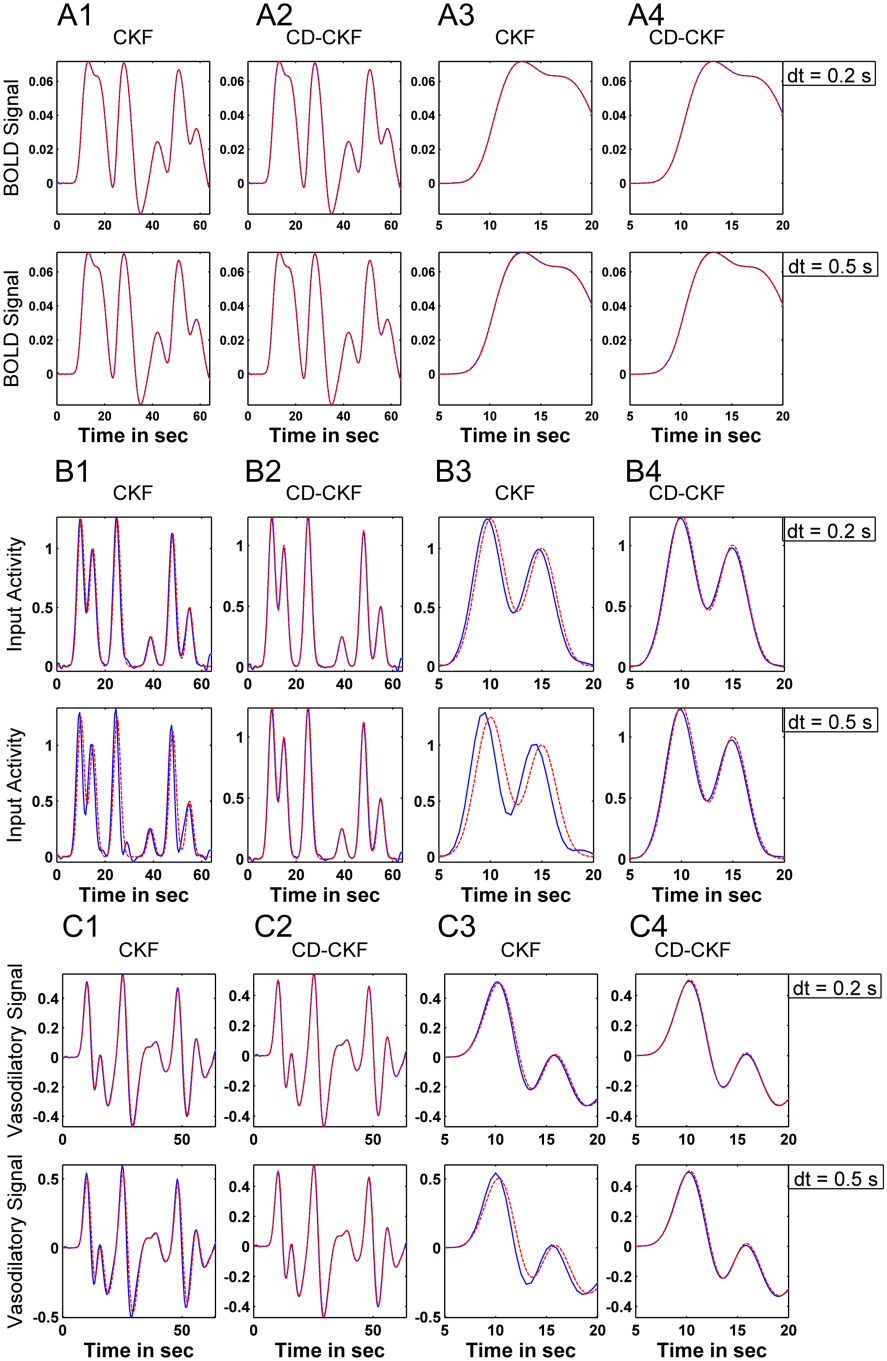
Performance of the Cubature Kalman filters (with backward smoothers) for estimating hemodynamic states from simulated BOLD signals under unknown NA inputs. A: BOLD signal and its CKF (left) and CD-CKF (right) estimates for sample interval *dt* = 0.2, and 0.5 *sec* (top and lower rows, respectively). In all figures, simulated signals are in red and estimates in blue. Shaded blue regions correspond to 95% confidence intervals (100 simulations) which are extremely tight around the mean value. B: NA input for CKF (B1) and CD-CKF (B2), which are enlarged in B3, B4 respectively. C: vasodilatory signal for different time *dt* = 0.2, and 0.5 *sec*.

We tested the performance of CKF and the hybrid CD-CKF in performing blind input deconvolution under two scenarios of, first, unknown neural activity (NA) input and, second, unknown NA inputs and model parameters (see Methods section). Fig 16A shows the simulated BOLD signals (red trace) and estimated BOLD signal (overlapping blue trace) for both filters under the first scenario (unknown NA input) for two time steps (*dt* = 0.2, 0.5 *sec*). The corresponding estimated NA input, which was obtained after a smoothed backward pass of both filters (Cubature smoother, see Methods section), is shown in Fig 16B (red trace: true input, blue trace: estimated input). It is noted here that the CKF produced inaccurate estimates of the input at larger time steps (Fig 16B1 and 16B3, bottom). More importantly, the CKF was unable to accurately localize the time of occurrence of the NA input (input timing) for both time steps (Fig 16B1 and enlarged plots in Fig 16B3). On the other hand, the CD-CKF produced more robust estimates of both the magnitude and input timing dynamics for the two sampling times (*dt* = 0.2, 0.5 *sec*) (Fig 16B2 and enlarged plots in Fig 16B4). Finally, Fig 16C shows the estimates of one hidden state, the vasodilatory signal (*s*), which exhibits similar performance limitations of the CKF (in terms of signal shape and timing inaccuracy) when compared to the CD-CKF.

**Fig 17:**
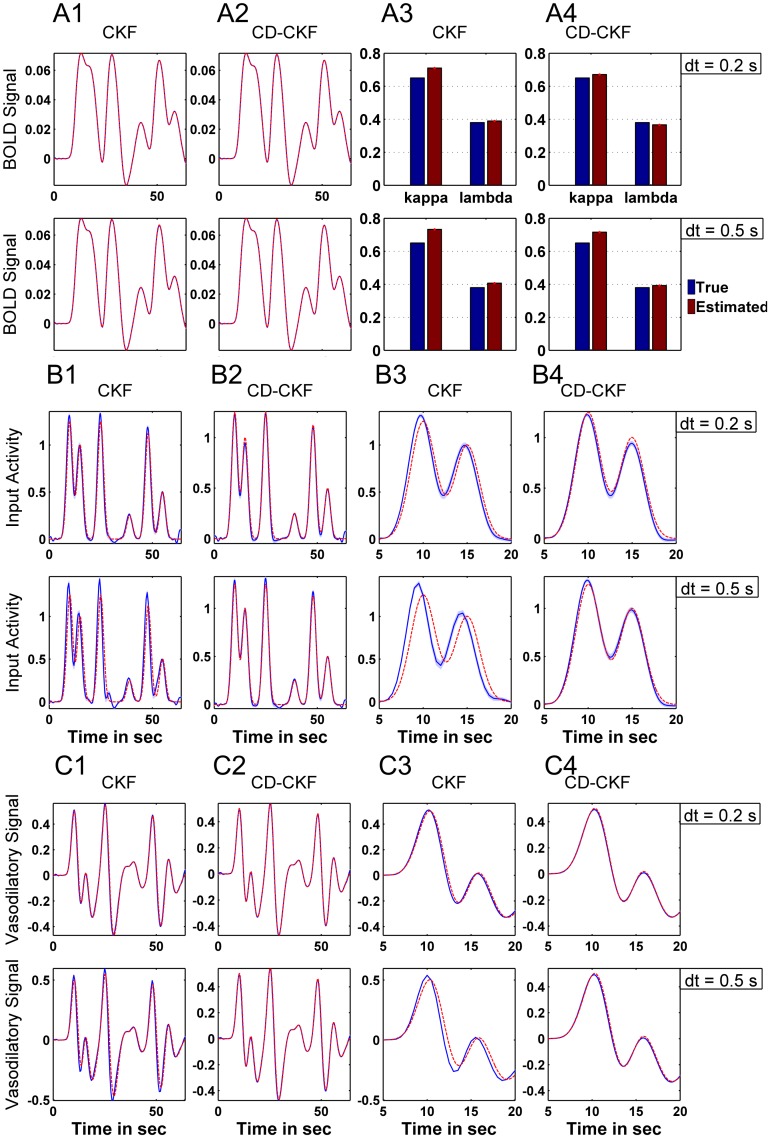
Performance of the Cubature Kalman filters (with backward smoothers) for estimating hemodynamic states from simulated BOLD signals under unknown NA inputs and two unknown parameters. A1-A2: BOLD signal and its CKF (left) and CD-CKF (right) estimates for sample interval *dt* = 0.2, and 0.5 *sec* (top and lower rows, respectively). A3-A4: Estimated parameters (rate of signal decay *κ* and rate of feedback regulation *λ*). B: NA input for CKF (B1) and CD-CKF (B2), which are enlarged in B3, B4 respectively. C: Estimated vasodilatory signal.

The estimation accuracy for the second scenario (unknown NA input and unknown model parameters) is shown in Fig 17. Again, and while the BOLD signal is fitted properly with both CKF and CD-CKF, the timing accuracy of the input (Fig 17B) and hidden states (Fig 17C) continues to be better for the hybrid filter under the two time steps (*dt* = 0.2, 0.5 *sec*). The average values of the two parameters estimates show performance of the two filters (Fig 17A3 and 17A4). To gain more understanding, the normalized MSE values obtained for both CD-CKF and CKF are averaged over 100 Monte-Carlo runs of the two scenarios at different sampling rates are given in Table 8. A clear superior performance of the CD-CKF is seen in all the cases for the input, state and parameter estimates.

**Table 8:** Normalized MSE values averaged over 100 Monte-Carlo runs of the hemodynamic estimation for both CKF and CD-CKF filters.

			*dt* in *sec*	
			0.5	0.2
First scenario	CKF	MSE of the states	14 e^- 4^	1.8 e^- 4^
		MSE of the input	169 e^- 4^	31 e^- 4^
	CD-CKF	MSE of the states	0.62 e^- 4^	0.13 e^- 4^
		MSE of the input	5.7 e^- 4^	2.8 e^- 4^
Second scenario	CKF	MSE of the states	13 e^- 4^	1.8 e^- 4^
		MSE of the input	148 e^- 4^	29 e^- 4^
		MSE of the parameters	109 e^- 4^	48 e^- 4^
	CD-CKF	MSE of the states	0.62 e^- 4^	0.14 e^- 4^
		MSE of the input	6.04 e^- 4^	7.7 e^- 4^
		MSE of the parameters	58 e^- 4^	14 e^- 4^

### Joint neuronal-hemodynamic model

We simulated the neuronal-hemodynamic model for four seconds in which a 2-second train of 200 *ms* pulses (50% duty cycle) is delivered as excitation to the neuronal model. The hemodynamic model is subsequently driven by the firing rate of the infra-granular layer activity and both models were under the influence of additive white Gaussian noise. We assume a noisy BOLD observation signal to be collected at different sampling rates (*dt* = 2, 4, 8, 10 *ms*). Despite the fact that real BOLD signals has much slower sampling rate (in seconds), we present this proposed model solely to explore the benefits of the CD-CKF over the CKF when measurements have a slower rate than required by the dynamics of the underlying system.

We consider the continuous time system as driven by a Wiener noise process for a given SNR. For the purpose of simulation, we adopt a time step of 0.1 *ms* in IT-1.5 discretization method in order to generate the observation BOLD signal. We then artificially resample this signal at different sampling intervals (*dt* = 2, 4, 8, 10 *ms*) and make it available as measurement data for the two filters. Overall, we perform a total of 20 independent Monte-Carlo simulations in which we assess the performance of both CD-CKF and CKF at different sampling rates. It is worth mentioning that *dt* was chosen up to *dt* = 10 *ms* since the CKF starts to diverge and fail for larger values whereas the CD-CKF continues to converge up to sampling interval *dt* = 45 *ms* (given that the CD-CKF sampling interval *dt* is divided into *m* steps of length *δ*, where *δ = dt*/*m*, and *m* is taken to equal 10).

**Fig 18:**
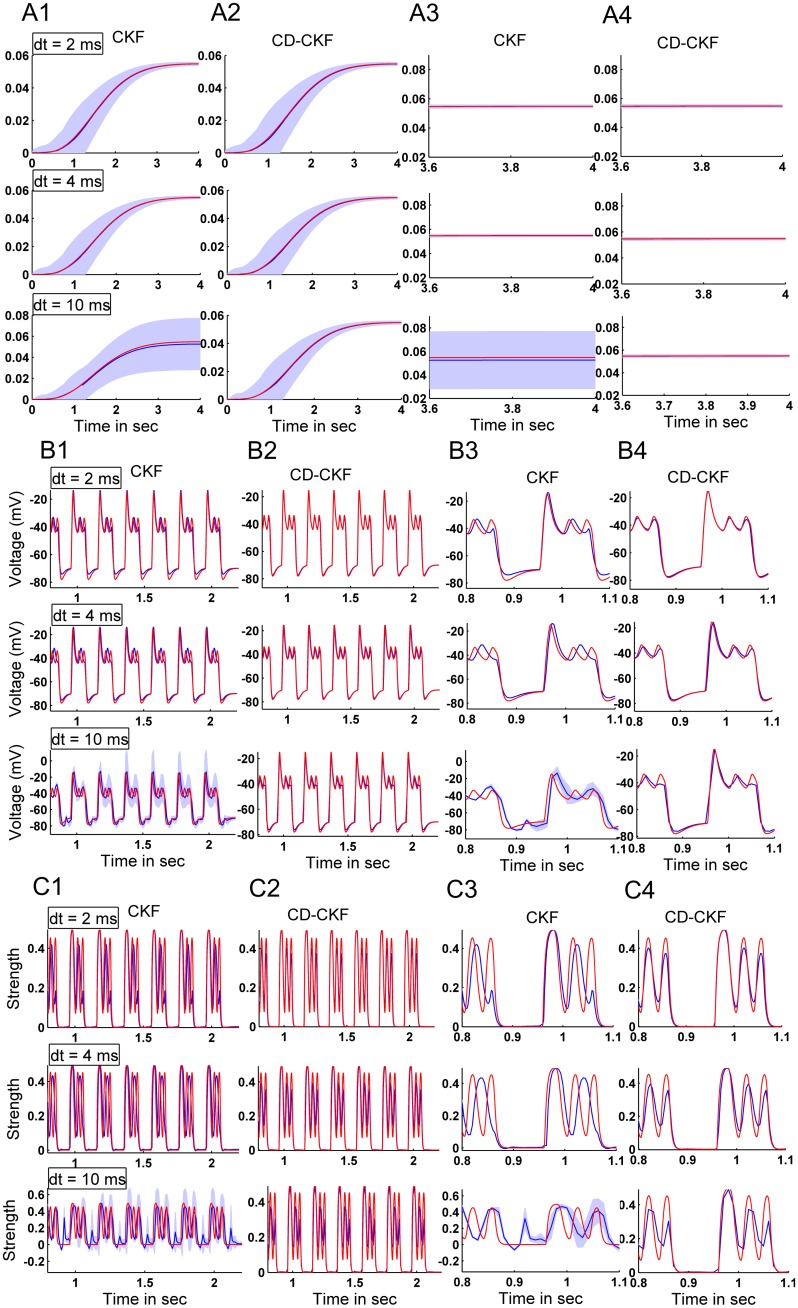
Comparison of the investigated Kalman filters for neural estimation from simulated fMRI signals. A: BOLD signal and its CKF (left) and CD-CKF (right) estimates for sample interval *dt* = 2, 4, and 10 *ms* (top, middle and lower rows, respectively). In all figures, simulated signals are in red and estimates in blue. Shaded blue regions correspond to 95% confidence interval (20 simulations). B: Infra-granular membrane potential for CKF (B1) and CD-CKF (B2), which are enlarged in B3, B4 respectively. C: Granular excitatory conductance for different time samples (*dt* = 2, 4, and 10 *ms*).

Fig 18A shows the simulated and estimated BOLD signal for increasing time steps (*dt* = 2, 4 and 10 *ms*). For the observed output, we note that while CKF and CD-CKF are able to predict the average signal (blue line) that is close to the simulated BOLD signal, the CKF exhibited large variation in performance across the MC simulations (shaded blue area, wider 95% confidence interval in subplot Fig 18A1 and enlarged Fig 18A3). The CD-CKF predicted output, on the other hand, was fairly unaffected up to the largest interval reported.

Among the estimated hidden neuronal states, we present samples that are increasingly farther removed away from the BOLD observation. In particular, we consider the membrane potential in the infra-granular layer (*V*^(3)^, cf. Fig 1, whose firing output feeds the hemodynamic model) and the excitatory conductance in the granular layer ($g_{E}^{\left(1\right)}$ which is one step further removed from neuronal output). These are shown in Fig 18B and 18C. We first analyze the CKF performance. We here note that the CKF-based estimate has lower variance at small *dt* = 2 *ms* (more consistent performance across MC simulations) for both membrane voltage and granular conductance. The mean value, however, does not track the fast ripple dynamics voltage for the membrane (Fig 18B3) and particularly for the conductance (Fig 18C3). With increasing *dt*, this tracking performance becomes worse (*dt* = 4 *ms*, middle plots) and the variance is noted to increase for the CKF. At the largest *dt* = 10 *ms*, the CKF estimate has larger variances with time. This indicates tendency to lose track of the actual states across trials, and accordingly to perform worse particularly for the conductance (Fig 18C3, lower plot) where the dynamics are nearly lost with very large errors across trials. In contrast, the CD-CKF performance was fairly unaffected by the increase in time step and was largely able to recover the fast ripple dynamics for *dt* = 2, 4 *ms*, with slight decrease in performance for *dt* = 10 *ms* (Fig 18B2, 18B4, 18C2 and 18C4). These estimates were furthermore consistent across the MC simulations as implied by the very tight confidence interval around the mean.

Table 9 lists the normalized MSE values averaged over 20 Monte-Carlo runs of CD-CKF and CKF at different sampling rates. It is clear that the CD-CKF outperformed the CKF for all sampling rates in terms of lower MSE values and lower variance. It is noted that for *dt* = 10 *ms* although the MSE value of the CKF is relatively acceptable compared to those of lower sampling rates, the high variance makes the estimation unreliable as seen in Fig 18.

**Table 9:** Normalized MSE values averaged over 20 Monte-Carlo runs of CKF and CD-CKF filters for white noise with the corresponding variance.

		*dt* in *ms*			
		2	4	8	10
CKF	MSE	0.009	0.012	0.013	0.035
	variance	3.4e^-7^	3.75 e^-7^	1.05 e^-6^	0.006
CD-CKF	MSE	0.003	0.003	0.006	0.02
	variance	3.7 e^-7^	3.9 e^-7^	5.7 e^-7^	2.89 e^-7^

**Fig 19:**
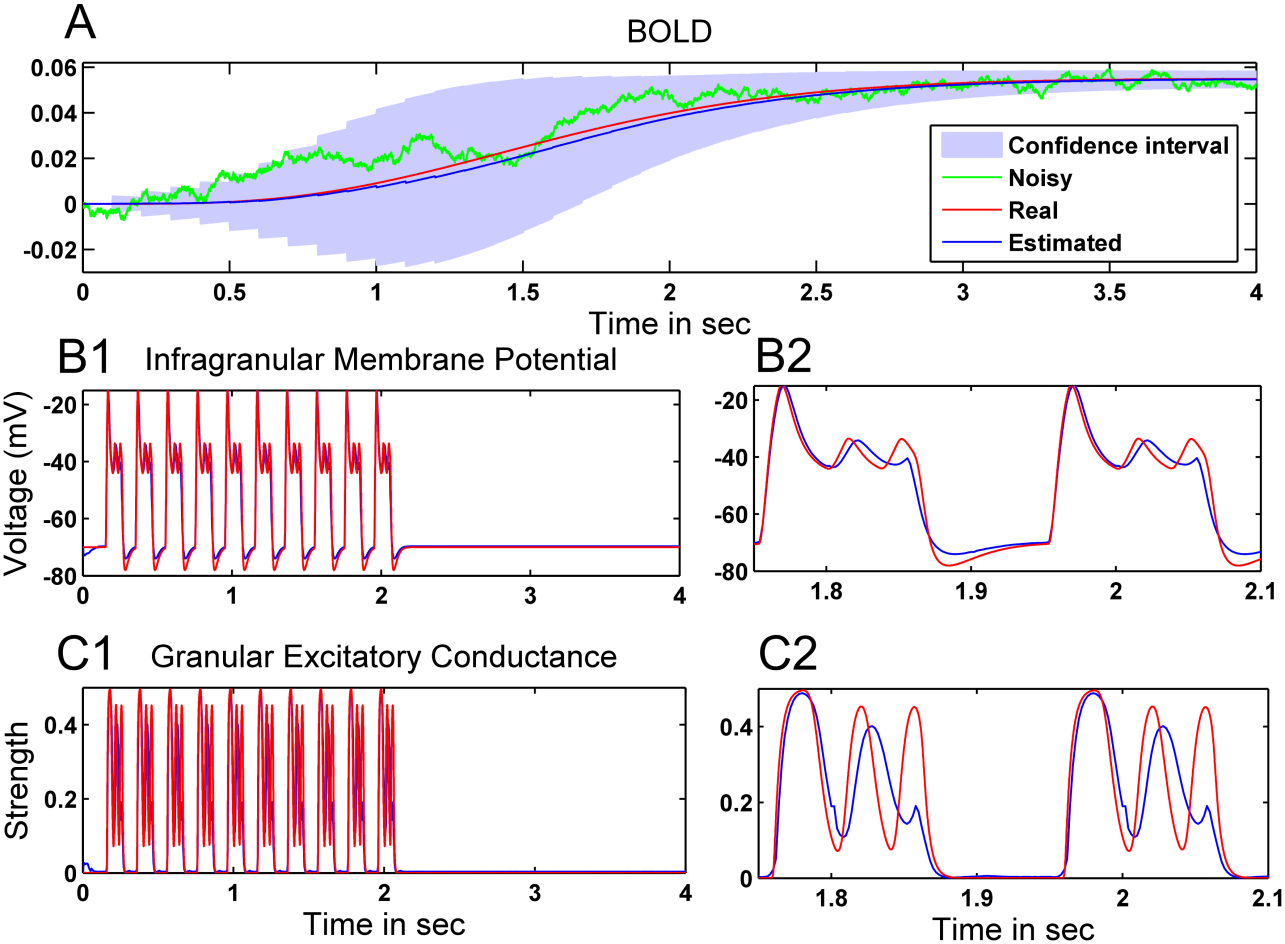
Performance of the CD-CKF for estimating neural firing dynamics from simulated BOLD signals under known neural inputs. A: One recorded sample (noisy), clean and estimates BOLD. Shaded blue regions correspond to 95% confidence interval (20 simulations). B1: Clean and estimated membrane potential (red and blue traces, respectively), enlarged in B2.). C1: Clean and estimated excitatory conductance (red and blue traces, respectively), enlarged in C2.

Finally, we present in Fig 19 preliminary simulations on using the CD-CKF to estimate the fine-grained neural electrical activity (scale of ~20 *ms*) from a noisy BOLD measured signal (scale of ~1 *sec*) and known input to the neural population. Starting with a simulated BOLD signal that is collected over 1 second intervals (repeat time = 1*sec*), we produced linear interpolations spaced at 100 *ms* intervals between successive samples. This resampled signal subsequently constitutes an effective discrete-time observation to be used by the CD-CKF. In the filter time-update step between observations, we utilized *m* = 50(*δ* = *dt*/*m*) subintervals (thus producing an IT-1.5 approximation of the integral every 2 *ms*). Finally, we assume a known neural input signal, as a train of 100 *ms* pulses repeating every 200 *ms* for 2 seconds. Fig 19A shows an example simulated noisy BOLD signal used as observation (green line), the noise-free clean signal (red line), the estimated mean BOLD signal over 20 Monte Carlo simulations (blue line), and the corresponding 95% confidence interval. The estimate is mostly very close to the clean trace with the error largest during the transient period (where the filter is tracking the state changes). Fig 19B1, 19B2, 19C1 and 19C2 again show the membrane potential and the excitatory conductance, respectively. In the enlarged traces in Fig 19B2 and 19C2, we note that the time update (which starts from interpolated discrete observations) is able to track the salient neural dynamics despite the presence of noisy BOLD observations.

## Conclusion and discussion

In this paper, we analyze the performance of two relatively novel nonlinear Bayesian estimation techniques, namely the discrete Cubature Kalman filter and the hybrid Continuous–Discrete Cubature Kalman filter, that carry significant promise in efficiently and recursively estimating causal nonlinear models of hidden continuous random processes using a limited set of indirect observations. Examples of such processes are dispersed throughout biological phenomena, and are especially abundant and relevant in the field of Neuroscience. We here focus on the two problems of (a) estimating neural firing and intracortical conductances from direct real-time observations such as electric field potential (or EEG), and (b) estimating neural activity drive and hemodynamic parameters from indirect time-sampled observations such as BOLD signals (or fMRI).

Our results show that the explicit consideration of the continuous nature of the underlying biological process can (1) provide a significant improvement in the accuracy of the estimates and (2) allow for a wider range of noise processes that are commonly thought to adversely affect the applicability of Gaussian-based techniques such as the Kalman filter.

First, we used simulated noisy electric potential recordings to assess the accuracy of discrete and hybrid Kalman techniques in estimating the cortical neural firing rates. We estimated these rates as hidden realizations of the continuous time process that is governed by nonlinear dynamics and subjected to in vivo random noise. We here chose a popular model of the laminar profile of cortical neural population activity among a multitude of candidate models available in the literature and that principally have similar nonlinearities (sigmoidal nature) and continuous dynamics (neural membrane equations). We have addressed, using multiple Monte-Carlo simulation runs, the accuracy of the hidden states obtained with the two tested Kalman filtering techniques under different assumptions on (i) the data sampling rate at which the observations are obtained, (ii) the signal-to-noise ratio in the observations, and (iii) the structure of the modeled process noise that additively affect the hidden process dynamics. We quantified the performance of a given filter in terms of the common mean square error (MSE) in the estimate (averaged over 100 Monte-Carlo simulations) and two devised measures of accuracy of the obtained estimates: the total fraction of time (PI) and the amount of deviation (LI) that an estimate is farther away than a threshold percentage (20%) from the true states.

Second, we simulated BOLD signal recordings to assess the ability of the two Kalman techniques in (i) deconvolving the input neural activity and (ii) estimating model parameters. We again chose a popular hemodynamic model particularly to benchmark the accuracy obtained here against state-of-the art estimation results that are reported in the neuroimaging literature and that used these models. We here focused on the superior ability of the hybrid filter (CD-CKF) in estimating the amplitude and, critically, the timing of the neural input.

Finally, we simulated BOLD signal recordings as obtained from a joint neural-hemodynamic model to study the ability of both techniques to estimate neural firings (under known inputs) from these indirect observations. We presented a primary example on the unprecedented ability of a CD-CKF estimator to obtain fine-grained neural firing profiles (~ 20 *ms*) from noisy BOLD low time resolution (~ 1 *sec*) observations.

We summarize our key findings as follows:

### Performance of the two filters under Gaussian process noise

For the two cases of white (independent) and colored (dependent) Gaussian noise structures (Figs 4–7), state estimates that were obtained with either the CKF and CD-CKF techniques expectedly improved as the sampling time step size *dt* is decreased and the observation quality (SNR) is increased. In comparing the performance of both filters (in terms of MSE ratios over 100 simulations), the estimation accuracy for the cases of intermediate time step sizes (*dt* = 1–2 *ms*) was (i) comparable under low SNR level but (ii) higher for the CD-CKF under larger SNR levels. Importantly, the CD-CKF estimation accuracy was significantly higher in the simulated cases of both very small and very large time steps (*dt* = 0.1–0.5 *ms*, *dt* = 4–8 *ms*, respectively) regardless of noise levels. The obtained results were consistent regardless whether the observations were obtained by simulating the system using the IT-1.5 or the LL discretization methods.

### Effect of observation noise level

An improvement in the signal quality (higher SNR) expectedly resulted in an increase in the accuracy of the estimates for both filtering techniques. Still, the rate of increase was significantly larger when using the CD-CKF, particularly for lower SNR values. This result was consistent regardless of the noise structure and the sampling interval tested.

### Effect of sampling time step

For all the noise structures tested, the state estimation accuracy (MSE) obtained when using the CKF and CD-CKF was generally comparable for intermediate step sizes (*dt* = 2 – 4*ms*) particularly under low SNR levels (Figs 9–15). At the largest sampling interval, the estimates obtained with CD-CKF were significantly better than those obtained with the CKF, regardless of the observation noise levels and structure. Importantly, collecting even more frequent samples, or reducing the sampling interval below *dt* = 1 *ms*, resulted in a continuous improvement in the performance of the CD-CKF but no improvement in that of the CKF performance which exhibited a “plateau” in its accuracy.

### Effect of process noise structure

The CD-CKF was more robust than the CKF against a wide range of additive noise structures that violated the Gaussianity assumption inherent to Kalman filtering techniques. The CD-CKF outperformed the CKF in all the cases of non-Gaussian additive noise considered. Furthermore, the CD-CKF performance at high SNR levels was less dependent on the actual noise structure and approached that of Gaussian noise. In other words, the CD-CKF was able to utilize the decrease in observation uncertainty (noise power) to adaptively correct the state estimates. Among all the tested discrete random noise structures, low frequency noise constituted the most challenging structure for both filters, possibly since the power of this specific signal is more concentrated within the frequency range of the system output (leading to effective lower SNR levels). In the case of neural systems, this is a less likely scenario since the electric potential recordings (i.e. observations or output), are often noted to have lower frequency range compared to in-vivo fluctuations in the firing rate or synaptic conductances (i.e. hidden process noise).

To summarize, the apparent gains made by using a CD-CKF over using a CKF in the estimation step are focused mainly in the following situations

1. The sampling interval is large. Here, the multiple prediction steps that the CD-CKF undertakes within the process model are able to better account of the uncertainty in the hidden states even when the noise structures violate Gaussianity assumptions. This was seen in both the detailed field potential study and briefly in the hypothetical hemodynamic modeling problem.
2. The sampling interval can be made increasingly small. Here, the approximation accuracy of the multiple prediction steps is able to better simulate the effect of the additive continuous process noise, particularly if the SNR values are large and regardless of the noise structure.
3. The effect of process noise on signal quality is minimized. In the context of neural systems, the process noise implicated in a Kalman setup is mainly background synaptic activity arriving onto a given neural population. Process noise attenuation, therefore, can be obtained by averaging across multiple trials of a given experiment, such as when recording cognition-related evoked potentials (e.g. somatosensory or visual).

Another important result is the ability of non-linear Kalman filtering techniques to overcome the limiting Gaussianity assumption on process noise structure in neural modeling. For the latter class of models, it is commonly assumed that the background noise impinging on local neural populations is the resultant of neuronal firing that is well approximated by a Poisson process. Current simulations demonstrated that the performance of both Kalman filters under Poisson process noise showed mild deterioration compared with that under Gaussian (white or colored) noise. In particular, the CD-CKF estimates under Poisson noise were very close to their counterparts under Gaussian noise. Furthermore, the quality of such CD-CKF estimates can be significantly improved by employing faster output sampling, a property that did not seem to hold for the CKF estimates.

### Hemodynamic model estimation

Estimating the neural activity underlying a recorded BOLD signal is a blind deconvolution problem that is confounded by the presence of nonlinearities in the hemodynamic process and time variation of the underlying neural activity. The results reported herein build on recent work that employed the CKF in estimating effective neural connectivity from fMRI data [32]. In the latter reference, the authors compared the performance of Cubature Kalman filtering to Dynamic Expectation Maximization (DEM) [49], and included a thorough discussion on the utility and advantages of using recursive nonlinear cubature Kalman filters.

In the current work, both the CKF and CD-CKF were able to estimate the overall profile of a low-frequency neural activity driving the hemodynamic model. However, only the CD-CKF provided an accurate profile at increased time steps. Critically, the CD-CKF with backward smoothing was able to provide an accurate time localization of the neural input. This occurred particularly since it explicitly accounts for the continuous dynamics over increasingly smaller interpolation steps of the low frequency observations. The ability to obtain accurate timing is of obvious importance to the whole series of studies that provide model-based estimates of the causal functional connectivity (directed information transfer) among brain areas using fMRI experiments [39, 58].

Finally, the joint neural-hemodynamic model attempts to go one step further by estimating all the states across the cascade of dynamic models with disparate temporal scales (neural firing dynamics at few milliseconds and BOLD signals at 0.5 to 1 second). It is seen that a CD-CKF smoother can still produce accurate estimates well beyond those obtained from discrete CKF estimators. The affirmative results obtained here are based on the assumption that the input to the neural population model is available and hence no input-deconvolution is needed. In fact, the latter is an ill posed problem particularly since the low-pass nature of BOLD measurements makes it theoretically impossible to reconstruct high frequency input traces in the absence of additional constraints on the nature of these inputs.

### Modeling accuracy

It is important to note here that the reported results are those of estimation and not modeling accuracy. In other words, the work implicitly assumes that the model structure chosen is inclusive of the dynamics of the underlying phenomenon. The modeling uncertainties are also assumed to satisfy the process and/or observation noise structures and levels discussed. The problem of model selection continues to be a central question whereby a series of candidate models are tested against available data. Notwithstanding the importance of the prior selection of a model structure, validating (or falsifying) a model depends critically on the estimation accuracy of its various parameters, inputs and states. Here, the selection is commonly assessed using variants of the estimation (mean square) error in the posterior estimate, possibly coupled with complexity criteria (Bayesian and Akaike information measures). The presented results were obtained for neural and hemodynamic models that are commonly used in the literature to illustrate the accuracy of the estimation as an integral part of an overall model design and selection experiment.

### Significance and implications

Other forms of Kalman filtering techniques have been applied in the area of neural modeling and connectivity estimation [28, 59, 60]. Specifically, the Unscented Kalman filter (UKF) is a nonlinear estimator that was employed in data assimilation is seizures [24, 61–64] and sleep dynamics [27]. Since its introduction, the cubature Kalman filter CKF has been noted to outperform the UKF both in terms of accuracy and numerical stability [2]. The current work suggests further refinements of the Kalman filtering theory in the area of neural modeling to include hybrid treatment of the continuous process equation and the discrete observations.

The presented work demonstrates a major improvement in estimation performance of a Kalman technique when the hidden dynamical process, such as neural activity, is explicitly treated as a continuous process within the time update of the filter. The power of this method becomes even more apparent when model inversion is attempted using observations that are obtained over disparate time scales. In particular, our simulations illustrate that as the sampling step size of a given recording is increased relative to the transient dynamics of the process, the ability to obtain estimates of parameters punctuating these specific dynamics becomes limited. First, in the case of electric potential recordings, the impact of fast dynamics, such the fast activity of AMPA or GABA-A synapses, cannot be clearly deciphered as the sampling interval increases beyond a few milliseconds. This situation is seen as particularly limiting for the CKF which, unlike the CD-CKF, does not incorporate an explicit treatment of the discrete observations. On the other hand, and because the CD-CKF integrates the impact of noise within the continuous dynamics during the time-update (prediction) step, the filter performance deteriorates marginally in estimating the hidden dynamics for sampling intervals that are quite large (*dt* = 4–8 *ms*) in relation to the speed of such dynamics (membrane time constant ~20 *ms*).

In the case of recordings obtained from other inherently slower modalities (e.g. fMRI, SPECT), the effect of the sampling step size becomes even more pressing when assessing the accuracy attainable under a CKF (or similar traditional discretization techniques). Indeed, CKF performance deteriorates when fitting neural population models (operating on the millisecond scale) based on a set of observations that (a) are obtained at much slower rates, and (b) are obtained from an aggregated measure of the neural activity that evolve with slower dynamics (such as oxygen metabolism). Here, the hybrid filter (CD-CKF) provides accurate estimates of the neural activity input profile as well as significant improvement in estimating the timing of the input activity. The latter is particularly useful in assessing the activation sequence of brain areas from fMRI data, a cornerstone tool in brain functional connectivity estimation.

We believe that these performance improvements gained by employing the hybrid CD-CKF are principally due to a major difference between the time update steps of the CKF and CD-CKF (that is, the prediction of the hidden states based on previous measurements and predictions). Specifically, the time update of the CD-CKF is divided into sub-intervals that propagates the predicted states through the process function at a higher sampling rate than that of the collected observations, whereas the time update of the CKF propagates the predicted states at the same sampling rate of collected observations. The frequent time-updates of the CD-CKF also allow for closer approximation of non-Gaussian process noise, further boosting the accuracy of the prediction for a wider class of environmental noise. Therefore, at a given sampling rate, the CD-CKF will always have more accurate predictions propagated from the time update to the measurement update when compared to the CKF, and hence will provide more accurate estimates. Critically, in applications where the time scale of the available measurements is limited by the modality (e.g. fMRI), the CD-CKF outperforms CKF since it explicitly accounts for the significant under-sampling of the faster dynamics of the underlying process (e.g. neural activity).

Finally, the time-recursive nature of Kalman filtering (particularly its ability to adaptively adjust tracking various model parameters) emphasizes the relevance of the reported results to modeling extensions in cases where the internal model parameters could vary with time, such as during synaptic plasticity and activity modulation across vigilance states, and with externally manipulations (e.g. transcranial stimulation).

## Appendix

### Cubature Kalman filter

Cubature Kalman filter is a nonlinear filter designed for hidden state estimation from nonlinear dynamic system with additive noise. The nonlinear dynamic system is described by a state-space model comprising a process and measurement equation. The process equation describes the continuous dynamics of the system and it is expressed by a continuous stochastic differential equation. The behavior of the dynamic system is observed through noisy measurements acquired at discrete time points and it is described by a discrete difference equation.

The state-space model is formulated as:

$$\begin{array}{llll} \text{Process Equation}: & dx\left(t\right) & = & f\left(x\left(t\right),t\right)dt+\sqrt{Q}d\beta \left(t\right)\\ \text{Measurement Equation}: & z_{k} & = & h\left(x_{k},k\right)+w \end{array}$$ (1)

Where $x\left(t\right)\;\;\in \;\;{\mathbb{R}}^{n}$ is the state of the dynamic system at time t, $z_{k}\;\in \;{\mathbb{R}}^{d}$ is the measurement at time *t*_*k*_, $f:\;{\mathbb{R}}^{n}\times \mathbb{R}\to {\mathbb{R}}^{n}$ is the drift coefficient, $h:\;{\mathbb{R}}^{n}\times \mathbb{R}\to {\mathbb{R}}^{d}$ is the measurement function, $\beta \left(t\right)\;\in \;{\mathbb{R}}^{n}$ is a standard Wiener process assumed to be independent of states and measurement noise,. $Q\;\in \;{\mathbb{R}}^{n\times n}$ is the diffusion coefficient, $w_{k}\;\in \;{\mathbb{R}}^{d}$ is a vector of random Gaussian measurement noise with zero mean and covariance *R*_*k*_.

Two methodologies are proposed to deal with the continuous process equation:

1. Discretize the SDE using Local Linearization (LL) method [65, 66], this will transform the state-space model to a pair of difference equations, and then apply the regular CKF.
2. Use the CD-CKF which discretizes the SDE using Itô-Taylor expansion of order 1.5 (IT 1.5).

### 1. CKF

Local Linearization method consists of transforming a nonlinear SDE to a linear SDE by applying the truncated Itô-Taylor expansion to the drift coefficient *f*(*x*(*t*), *t*), then evaluate the analytical solution of the resulting linear SDE and finally approximate the Itô’s integral in the obtained solution by means of the composite Trapezoidal rule [65]. The resultant discrete difference equation will be as follows:

$$x_{k}=f_{d}\left(x_{k-1},k-1\right)+v_{k-1}$$

Where $v_{k-1}\;\in \;{\mathbb{R}}^{n}$ is a vector of random Gaussian noise with zero mean and covariance *V*_*k*−1_, and

$$f_{d}\left(x_{k-1},k-1\right)\approx x_{k-1}+J_{k-1}^{-1}\left[exp\left(J_{k-1}\Delta t\right)-Ι\right]f\left(x_{k-1},k-1\right)$$

$J_{k}$ is the Jacobian of *f* and Δ*t* is the time interval between samples.

The state-space model (1) becomes:

$$\text{Process Equation}:\;x_{k}=f_{d}\left(x_{k-1},k-1\right)+v_{k-1}$$ (2)

$$\text{Measurement Equation: }z_{k}=h\left(x_{k},k\right)+w_{k}$$ (3)

Both CKF and CD-CKF are based on Bayesian filtering paradigm under Gaussian domain, in which the posterior density of the state provides a complete statistical description of the state at that time [8].

The CKF includes two steps:

1. Time update: compute the predicted density $p\left(x_{k}|z_{1:k-1}\right)\sim N\left({\hat{x}}_{k|k-1},P_{k|k-1}\right)$. Where:
  $$\begin{array}{ll} {\hat{x}}_{k|k-1} & =E\left[x_{k}|z_{1:k-1}\right]=E\left[f_{d}\left(x_{k-1},k-1\right)|z_{1:k-1}\right]\\ & =\int f_{d}\left(x_{k-1},k-1\right)p\left(x_{k-1}|z_{1:k-1}\right)dx_{k-1}\\ & =\int f_{d}\left(x_{k-1},k-1\right)\times N\left(x_{k-1};{\hat{x}}_{k-1|k-1},P_{k-1|k-1}\right)dx_{k-1} \end{array}$$ (4)
  $$\begin{array}{ll} P_{k|k-1} & =E\left[\left(x_{k}-{\hat{x}}_{k|k-1}\right){\left(x_{k}-{\hat{x}}_{k|k-1}\right)}^{T}|z_{1:k-1}\right]\\ & =\int f_{d}\left(x_{k-1},k-1\right)f_{d}{\left(x_{k-1},k-1\right)}^{T}\times N\left(x_{k-1};{\hat{x}}_{k-1|k-1},P_{k-1|k-1}\right)dx_{k-1}\\ & \;-{\hat{x}}_{k|k-1}{\hat{x}}_{k|k-1}^{T}+V_{k-1} \end{array}$$ (5)
2. Measurement update: compute the posterior density $p\left(x_{k}|z_{1:k}\right)\sim N\left({\hat{x}}_{k|k},P_{k|k}\right)$.
  The filter likelihood density is assumed to be Gaussian:
  $$p\left(z_{k}|z_{1:k-1}\right)\sim N\left(z_{k};{\hat{z}}_{k|k-1},P_{zz,k|k-1}\right)$$
  where the predicted measurement:
  $${\hat{z}}_{k|k-1}=\int h\left(x_{k},k\right)\times N\left(x_{k};{\hat{x}}_{k|k-1},P_{k|k-1}\right)dx_{k}$$ (6)
  and the associated covariance
  $$\begin{array}{ll} P_{zz,k|k-1} & =\int h\left(x_{k},k\right)h{\left(x_{k},k\right)}^{T}\times N\left(x_{k};{\hat{x}}_{k|k-1},P_{k|k-1}\right)dx_{k}\\ & \;-{\hat{z}}_{k|k-1}{\hat{z}}_{k|k-1}^{T}+R_{k} \end{array}$$ (7)
  The cross-covariance between the state and the measurement is given by:
  $$P_{xz,k|k-1}=\int xh{\left(x_{k},k\right)}^{T}\times N\left(x_{k};{\hat{x}}_{k|k-1},P_{k|k-1}\right)dx_{k}-{\hat{x}}_{k|k-1}{\hat{z}}_{k|k-1}^{T}$$ (8)
  Thus, the conditional Gaussian density of the joint state and the measurement can be written as:
  $$p\left({\left[\begin{array}{cc} x_{k}^{T} & z_{k}^{T} \end{array}\right]}^{T}|z_{1:k-1}\right)\sim N\left(\left[\begin{array}{c} {\hat{x}}_{k|k-1}\\ {\hat{z}}_{k|k-1} \end{array}\right],\left[\begin{array}{cc} P_{k|k-1} & P_{xz,k|k-1}\\ P_{xz,k|k-1}^{T} & P_{zz,k|k-1} \end{array}\right]\right)$$
  From which the posterior density $p\left(x_{k}|z_{1:k}\right)\sim N\left({\hat{x}}_{k|k},P_{k|k}\right)$ is computed on the receipt of a new measurement *z*_*k*_, where:
  $${\hat{x}}_{k|k}={\hat{x}}_{k|k-1}+{Κ}_{k}\left(z_{k}-{\hat{z}}_{k|k-1}\right)$$
  $$P_{k|k}=P_{k|k-1}-{Κ}_{k}P_{zz,k|k-1}{Κ}_{k}^{T}$$
  $${Κ}_{k}=P_{xz,k|k-1}P_{zz,k|k-1}^{-1}$$

### 2. CD-CKF

Applying the Itô-Taylor expansion of order 1.5 to the process equation in state-space model (1) over the time interval (*t*, *t* + *δ*) yields [2]:

$$x\left(t+\delta \right)=f_{d}\left(x\left(t\right),t\right)+\sqrt{Q}w+\left(Lf\left(x\left(t\right),t\right)\right)y$$ (9)

Where:

$$f_{d}\left(x\left(t\right),t\right)=x\left(t\right)+\delta f\left(x\left(t\right),t\right)+\frac{1}{2}\delta^{2}\left(L_{0}f\left(x\left(t\right),t\right)\right)$$

$L_{0}$ and L are two differential operators:

$$L_{0}=\frac{\partial }{\partial t}+\sum_{i=1}^{n}f_{i}\frac{\partial }{\partial x_{i}}+\frac{1}{2}\sum_{j=1}^{n}\sum_{p=1}^{n}\sum_{q=1}^{n}{\sqrt{Q}}_{pj}{\sqrt{Q}}_{qj}\frac{\partial^{2}}{\partial x_{p}\partial x_{q}}$$

Lf is a square matrix having elements $L_{j}f_{i}\;\left(i,j=1,\ldots ,n\right)$

$$L_{j}f_{i}=\sum_{k=1}^{n}{\sqrt{Q}}_{kj}\frac{\partial f_{i}}{\partial x_{k}}$$

(***w***, ***y***) is a pair of correlated n-dimensional Gaussian random variables, which can be generated from a pair of independent n-dimensional standard Gaussian random variables (*u*_1_, *u*_2_) as follows:

$$w=\sqrt{\delta }u_{1}$$

$$y=\frac{1}{2}\delta^{\frac{3}{2}}\left(u_{1}+\frac{u_{2}}{\sqrt{3}}\right)$$

$$E\left[ww^{T}\right]=\delta tI_{n}$$

$$E\left[wy^{T}\right]=\frac{1}{2}\delta^{2}I_{n}$$

$$E\left[yy^{T}\right]=\frac{1}{3}\delta^{3}I_{n}$$

1. Time update: compute the predicted density $p\left(x_{k+1}|z_{1:k}\right)\sim N\left({\hat{x}}_{k+1|k},P_{k+1|k}\right)$.
  $$\begin{array}{ll} \hat{x}\left(t+\delta \right) & =E\left[f_{d}\left(x\left(t\right)\right)|z_{1:k}\right]\\ & =\int f_{d}\left(x_{k},k\right)\times N\left(x_{k};{\hat{x}}_{k|k},P_{k|k}\right)dx_{k} \end{array}$$ (10)
  $$\begin{array}{ll} P\left(t+\delta \right) & =E\left[f_{d}\left(x\left(t\right)\right)f_{d}^{T}\left(x\left(t\right)\right)|z_{1:k}\right]+\frac{1}{3}\delta^{3}E\left[Lf\left(x\left(t\right),t\right){\left(Lf\left(x\left(t\right),t\right)\right)}^{T}\right]\\ & \;+\frac{1}{2}\delta^{2}\sqrt{Q}E\left[{\left(Lf\left(x\left(t\right),t\right)\right)}^{T}\right]+\frac{1}{2}\delta^{2}E\left[Lf\left(x\left(t\right),t\right)\right]{\sqrt{Q}}^{T}\\ & \;-\hat{x}\left(t+\delta \right){\left(\hat{x}\left(t+\delta \right)\right)}^{T}+\delta Q \end{array}$$ (11)
  To compute the predicted state and its error covariance more accurately at time *t*_*k*+1_, the sampling interval *T* is divided into *m* steps of length *δ*, where *δ* = (*t*_*k*+1_ − *t*_*k*_)/*m* = *T*/*m*.
  Let $x_{k}^{j}$ denotes *x*(*t*) at time *t* = *KT* + *jδ*, (1 ≤ *j* ≤ *m*), the statistics of *x*_*k*+1_ are given by:
  $${\hat{x}}_{k|k}^{j+1}=\int f_{d}\left(x_{k}^{j},KT+j\delta \right)\times N\left(x_{k}^{j};\;{\hat{x}}_{k|k}^{j},P_{k|k}^{j}\right)dx_{k}^{j}$$ (12)
  $$\begin{array}{ll} P_{k|k}^{j+1}\approx & \int f_{d}\left(x_{k}^{j},KT+j\delta \right)f_{d}^{T}\left(x_{k}^{j},KT+j\delta \right)\times N\left(x_{k}^{j};\;{\hat{x}}_{k|k}^{j},P_{k|k}^{j}\right)dx_{k}^{j}\\ & +\frac{1}{3}\delta^{3}Lf\left({\hat{x}}_{k|k}^{j},KT+j\delta \right){\left(Lf\left({\hat{x}}_{k|k}^{j},KT+j\delta \right)\right)}^{T}\\ & +\frac{1}{2}\delta^{2}\sqrt{Q}{\left(Lf\left({\hat{x}}_{k|k}^{j},KT+j\delta \right)\right)}^{T}+\frac{1}{2}\delta^{2}Lf\left({\hat{x}}_{k|k}^{j},KT+j\delta \right){\sqrt{Q}}^{T}\\ & -{\hat{x}}_{k|k}^{j}{\left({\hat{x}}_{k|k}^{j}\right)}^{T}+\delta Q \end{array}$$ (13)
  The predicted density is computed at *t*_*k*+1_ for *j* = *m*, $p\left(x_{k+1}|z_{1:k}\right)\sim N\left({\hat{x}}_{k|k}^{m},P_{k|k}^{m}\right)$ (i.e. $p\left(x_{k+1}|z_{1:k}\right)\sim N\left({\hat{x}}_{k+1|k},P_{k+1|k}\right)$).
2. Measurement update: perform normal discrete-time CKF update to compute the posterior density $p\left(x_{k+1}|z_{1:k+1}\right)\sim N\left({\hat{x}}_{k+1|k+1},P_{k+1|k+1}\right)$.

### 3. Third-Degree Cubature Rule

The Bayesian filter in the Gaussian domain reduces to the problem of how to compute integrals of the following form (Arasaratnam et al. 2010):

I(f)=∫f(x)×N(x;.,.)dx

Where *f*(.) is some nonlinear function.

The heart of cubature Kalman Filter is to numerically approximate this type of integrals by third-degree spherical-radial rule using an even set of 2*n* equally weighted symmetric cubature points ${\left\{\xi_{i},\omega_{i}\right\}}_{i=1}^{2n}$ (where *n* is the dimension of the state vector) [2]:

$$I\left(f\right)=\int f\left(x\right)\times N\left(x;\mu ,\Sigma \right)dx\approx \sum_{i=1}^{2n}\omega_{i}f\left(\mu +\sqrt{\Sigma }\xi_{i}\right)$$ (14)

Where:

$$\Sigma =\sqrt{\Sigma }{\sqrt{\Sigma }}^{T}$$

$$\omega_{i}=\frac{1}{2n}$$

$$\xi_{i}=\{\begin{array}{ll} \sqrt{n}e_{i} & i=1,\ldots ,n\\ -\sqrt{n}e_{i} & i=n+1,\ldots ,2n \end{array}$$

Using this numerical approximation of integrals,

1. the time update of the CKF becomes:
  $$\begin{array}{ll} {\hat{x}}_{k|k-1} & =\int f_{d}\left(x_{k-1},k-1\right)\times N\left(x_{k-1};{\hat{x}}_{k-1|k-1},P_{k-1|k-1}\right)dx_{k-1}\\ & =\frac{1}{2n}\sum_{i=1}^{2n}X_{i,k|k-1}^{*} \end{array}$$
  Where
  $$X_{i,k|k-1}^{*}=f_{d}\left({\hat{x}}_{k-1|k-1}+\sqrt{P_{k-1|k-1}}\xi_{i},k-1\right)$$
  $$\begin{array}{ll} P_{k|k-1} & =\int f_{d}\left(x_{k-1},k-1\right)f_{d}{\left(x_{k-1},k-1\right)}^{T}\times N\left(x_{k-1};{\hat{x}}_{k-1|k-1},P_{k-1|k-1}\right)dx_{k-1}\\ & -{\hat{x}}_{k|k-1}{\hat{x}}_{k|k-1}^{T}+V_{k-1}\\ & =\frac{1}{2n}\sum_{i=1}^{2n}X_{i,k|k-1}^{*}X_{i,k|k-1}^{*T}-{\hat{x}}_{k|k-1}{\hat{x}}_{k|k-1}^{T}+V_{k-1} \end{array}$$
  And the measurement update
  $${\hat{z}}_{k|k-1}=\int h\left(x_{k},k\right)\times N\left(x_{k};{\hat{x}}_{k|k-1},P_{k|k-1}\right)dx_{k}=\frac{1}{2n}\sum_{i=1}^{2n}Z_{i,k|k-1}$$
  Where
  $$Z_{i,k|k-1}=h\left({\hat{x}}_{k|k-1}+\sqrt{P_{k|k-1}}\xi_{i},k\right)$$
  and the associated covariance
  $$\begin{array}{ll} P_{zz,k|k-1} & =\int h\left(x_{k},k\right)h{\left(x_{k},k\right)}^{T}\times N\left(x_{k};{\hat{x}}_{k|k-1},P_{k|k-1}\right)dx_{k}\\ & -{\hat{z}}_{k|k-1}{\hat{z}}_{k|k-1}^{T}+R_{k}\\ & =\frac{1}{2n}\sum_{i=1}^{2n}Z_{i,k|k-1}Z_{i,k|k-1}^{T}-{\hat{z}}_{k|k-1}{\hat{z}}_{k|k-1}^{T}+R_{k} \end{array}$$
  The cross-covariance between the state and the measurement is given by:
  $$\begin{array}{ll} P_{xz,k|k-1} & =\int xh{\left(x_{k},k\right)}^{T}\times N\left(x_{k};{\hat{x}}_{k|k-1},P_{k|k-1}\right)dx_{k}-{\hat{x}}_{k|k-1}{\hat{z}}_{k|k-1}^{T}\\ & =\frac{1}{2n}\sum_{i=1}^{2n}X_{i,k|k-1}Z_{i,k|k-1}^{T}-{\hat{x}}_{k|k-1}{\hat{z}}_{k|k-1}^{T} \end{array}$$
  Where
  $$X_{i,k|k-1}={\hat{x}}_{k|k-1}+\sqrt{P_{k|k-1}}\xi_{i}$$
2. the time update of the CD-CKF becomes:
  $$\begin{array}{ll} {\hat{x}}_{k|k}^{j+1} & =\int f_{d}\left(x_{k}^{j},KT+j\delta \right)\times N\left(x_{k}^{j};\;{\hat{x}}_{k|k}^{j},P_{k|k}^{j}\right)dx_{k}^{j}\\ & =\frac{1}{2n}\sum_{i=1}^{2n}X_{i,k|k}^{*\left(j+1\right)} \end{array}$$
  Where
  $$X_{i,k|k}^{*\left(j+1\right)}=f_{d}\left({\hat{x}}_{k|k}^{j}+\sqrt{P_{k|k}^{j}}\xi_{i},kT+j\delta \right)$$
  $$\begin{array}{ll} P_{k|k}^{j+1}\approx & \int f_{d}\left(x_{k}^{j},KT+j\delta \right)f_{d}^{T}\left(x_{k}^{j},KT+j\delta \right)\times N\left(x_{k}^{j};\;{\hat{x}}_{k|k}^{j},P_{k|k}^{j}\right)dx_{k}^{j}\\ & +\frac{1}{3}\delta^{3}Lf\left({\hat{x}}_{k|k}^{j},KT+j\delta \right){\left(Lf\left({\hat{x}}_{k|k}^{j},KT+j\delta \right)\right)}^{T}\\ & +\frac{1}{2}\delta^{2}\sqrt{Q}{\left(Lf\left({\hat{x}}_{k|k}^{j},KT+j\delta \right)\right)}^{T}+\frac{1}{2}\delta^{2}Lf\left({\hat{x}}_{k|k}^{j},KT+j\delta \right){\sqrt{Q}}^{T}\\ & -{\hat{x}}_{k|k}^{j}{\left({\hat{x}}_{k|k}^{j}\right)}^{T}+\delta Q \end{array}$$
  $$\begin{array}{l} \approx \frac{1}{2n}\sum_{i=1}^{2n}X_{i,k|k}^{*\left(j+1\right)}X_{i,k|k}^{*\left(j+1\right)}{}^{T}+\frac{1}{3}\delta^{3}Lf\left({\hat{x}}_{k|k}^{j},KT+j\delta \right){\left(Lf\left({\hat{x}}_{k|k}^{j},KT+j\delta \right)\right)}^{T}\\ \;+\frac{1}{2}\delta^{2}\sqrt{Q}{\left(Lf\left({\hat{x}}_{k|k}^{j},KT+j\delta \right)\right)}^{T}+\frac{1}{2}\delta^{2}Lf\left({\hat{x}}_{k|k}^{j},KT+j\delta \right){\sqrt{Q}}^{T}\\ \;-{\hat{x}}_{k|k}^{j}{\left({\hat{x}}_{k|k}^{j}\right)}^{T}+\delta Q \end{array}$$
  And the measurement update for CD-CKF is exactly the same as discrete-time CKF update.
